# Investigation on Human Carbonic Anhydrase IX and XII Inhibitory Activity and A549 Antiproliferative Activity of a New Class of Coumarinamides

**DOI:** 10.3390/ph18030372

**Published:** 2025-03-05

**Authors:** Davide Moi, Simone Carradori, Marialucia Gallorini, Noemi Mencarelli, Alberto Deplano, Andrea Angeli, Serena Vittorio, Claudiu T. Supuran, Valentina Onnis

**Affiliations:** 1 Department of Life and Environmental Sciences, Unit of Pharmaceuitical, Pharmacological and Nutraceutical Sciences, University of Cagliari, Cittadella Universitaria di Monserrato, I-09042 Monserrato, CA, Italy; davide.moi@unica.it (D.M.); alberto.deplano00@gmail.com (A.D.); 2 Department of Pharmacy, “G. d’Annunzio” University of Chieti-Pescara, Via dei Vestini 31, I-66100 Chieti, CH, Italy; simone.carradori@unich.it (S.C.); marialucia.gallorini@unich.it (M.G.); noemi.mencarelli@phd.unich.it (N.M.); 3 NEUROFARBA Department, Sezione di Scienze Farmaceutiche, University of Florence, Via Ugo Schiff 6, I-50019 Sesto Fiorentino, FI, Italy; andrea.angeli@unifi.it (A.A.); claudiu.supuran@unifi.it (C.T.S.); 4 Dipartimento di Scienze Farmaceutiche, Università degli Studi di Milano, Via Mangiagalli 25, I-20133 Milano, MI, Italy; serena.vittorio@unimi.it

**Keywords:** coumarins, amides, carbonic anydrase, enzyme inhibition, cytostatic activity

## Abstract

**Background**—Aggressive solid tumors are commonly characterized by both basic intracellular pH and acidic extracellular pH, which increase cell survival and proliferation. As carbonic anhydrases IX/XII are involved in this pH regulation, their inhibition is an appealing approach in cancer therapy, avoiding cancer cell survival and proliferation. Substituted coumarins are selective non-classical CA IX and CA XII inhibitors. **Methods**—In this study, new 7-hydroxycoumarinamides were synthesized and assayed for CA inhibition and antiproliferative activity. **Results**—All of the coumarinamides showed human CA IX and CA XII selective inhibition over the off-target CA I and CA II isoforms. Coumarin acts as a suicide inhibitor because its heterocyclic ring can be hydrolyzed by CA esterase activity to give the corresponding 2-hydroxycinnamic acid derivative which blocks the entrance of the active site. The 2-hydroxycinnamic acid derivatives deriving from the most potent and selective coumarinamides were docked into CA IX and XII to better understand the activity and selectivity against the two CA isoforms. The most active coumarinamides also produced a decrease of A549 cell proliferation and were able to arrest cells at the G1/S checkpoint. **Conclusions**—These results may open new perspectives for developing coumarin-based CA IX/XII inhibitors.

## 1. Introduction

**Figure 1:**
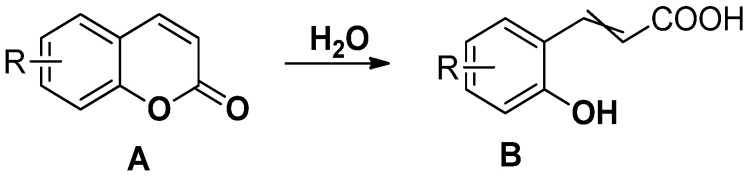
Hydrolysis of coumarin (**A**) to 2-hydroxycinnamic acid (**B**).

Solid cancers are characterized by high growth and spread, having several hypoxic regions as a result of this intense activity. The metabolic reprogramming/rewiring process is a crucial change in cancer cells, and it is strictly related with their overall survival [1]. Both oxygen and nutrient deficiencies trigger hypoxia-inducible factor (HIF) signaling which results in HIF1α stabilization. This transcriptional factor plays a crucial role in the expression and stabilization of several enzymes involved in the regulation of the tumor microenvironment [2]. Metabolism alteration is one of the most important modifications in solid tumors, followed by intra-and extra-cellular acid/base regulation [3]. In fact, the metabolic reprogramming of solid tumors results in the production of large amounts of acidic metabolites. The intracellular pH is precisely regulated and, in cancer cells, it becomes slightly basic as the extracellular pH increasingly decreases during cell progression, promoting both cell survival and proliferation. Intracellular alkaline pH may improve cell survival by the suppression of caspase activity and through modifications of mitochondria-dependent apoptosis mechanisms [4]. Furthermore, basic intracellular pH induces glycolytic activity, causing an increase in acid production and promoting extracellular acidification [5]. The acid extracellular environment modulates integrin-mediated cell–matrix adhesion and promotes the disruption of the extracellular matrix through the activation of several matrix metalloproteinases [6]. Consequently, the development of therapeutical strategies which affect these alterations related to metabolism and pH regulation may result in the reduction of the growth and aggression of cancer cells, also limiting the metastatic potential [7]. The acid/base regulation in cancer cells is tightly regulated by the combined activity of carbonic anhydrases (CAs) and different transmembrane proteins which mediate the removal of protons from the cytoplasm, generating the so called “transport metabolon” [8]. The transport metabolon is commonly composed of bicarbonate transporters and the cancer-related isoforms CA IX/XII or by CAs IX/XII and monocarboxylate transporters [9]. During recent years, much effort has been made to understand how to selectively inhibit these isoforms, so this understanding is currently an already proven target for cancer therapy. The importance of the cancer-related isoforms human *h*-CA IX and *h*-CA XII in cancer therapy has become increasingly evident due to the role of these enzymes not only in the survival of the cancer cells but also in the metastatic mechanisms. Metastatic lesions can be facilitated by *h*-CA IX through the formation of focal adhesion contacts and cell spreading, while the initial growth of metastasis takes advantage of *h*-CA IX-mediated pH regulation [10]. Additionally, it has been demonstrated that metastatic lesions are generated by cancer stem cells, showing the association between *h*-CA IX expression and this stem-like phenotype [11]. An understanding of the three-dimensional structures of catalytically active CAs is required to design new chemical entities that are able to selectively inhibit these cancer-related isoforms. In mammals, CAs are Zn^2+^-dependent enzymes in which the metal ion is placed at a 15 Å-deep bottom of the active site and is coordinated by three conserved histidine (H) residues (H94, H96, and H119) and a water molecule/hydroxide ion. Furthermore, because of the rapid exchange between CO_2_ and HCO_3^−^_, the active site is characterized by both hydrophobic and hydrophilic areas [12]. Several strategies for developing selective *h*-CA IX and *h*-CA XII inhibitors have already been reported [13], confirming the importance of primary sulfonamides as CA inhibitors. Sulfonamides, defined as classical CA inhibitors, coordinate the Zn^2+^ through their deprotonated form in the enzyme active site [14,15]. This mechanism is responsible for the high potency of sulfonamides, but also for their low selectivity towards the cytosolic *h*-CA I and *h*-CA II isoforms. On the other hand, different studies have proven the importance of substituted coumarins as selective non-classical *h*-CA IX and *h*-CA XII inhibitors [16,17,18,19,20]. Through X-ray crystallography, it has been found that a coumarin ring (**A**) can be hydrolyzed by CA esterase activity, affording the corresponding 2-hydroxycinnamic acid derivative (**B**) which blocks the entrance of the active site in a “suicidal inhibition” (Figure 1). Starting from these considerations, here we report a new class of 7-hydroxy coumarins characterized by the presence of substituted amides at the C-3 of the coumarin core. In this study we evaluated the new coumarins obtained by the introduction of substituted benzylamines, N-aryl piperazines and phenylethylamines, in order to correlate the different inhibitory activity with the flexibility of the molecule. This combination resulted in compounds with significant chemical diversity with respect to previously reported CA inhibitors, opening new perspectives for developing coumarin-based *h*-CA IX/XII inhibitors.

## 2. Results and Discussion

### 2.1. Chemistry

**Scheme 1:**
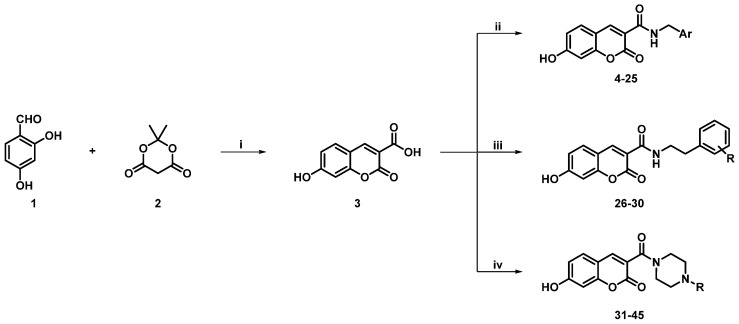
General synthetic procedure for 7-hydroxycoumarin amides **4**–**45**. Reagents and conditions are as follows: (i) CH_3_COONH_4_, water, reflux 15 h, 87% yield; (ii) substituted benzylamines, EDCI, HOBt, dry MeCN, r.t. 6 h, 44–92% yield; (iii) substituted phenylethylamines, EDCI, HOBt, dry MeCN, r.t. 6 h, 70–90% yield; (iv) substituted piperazines, EDCI, HOBt, dry MeCN, r.t. 6 h, 47–82% yield.

The synthetic pathway for obtaining coumarin amides **4**–**45**, reported in Scheme 1, started with a Knoevenagel condensation between the 4-hydroxy-salicylaldehyde (**1**) and the Meldrum’s acid (**2**) in water and under basic condition through ammonium acetate [21,22]. The obtained carboxylic acid **3** was coupled with appropriately substituted benzylamines, N-aryl piperazines and phenylethylamines by using 1-(3-dimethylaminopropyl)-3-ethylcarbodiimide hydrochloride (EDCI) in the presence of 1-hydroxybenzotriazole (HOBt) and in dry acetonitrile solution (MeCN) for piperazines and phenylethylamines, producing the desired amides **4**–**45** with yields of 44–92%.

### 2.2. Carbonic Anhydrase Inhibition Assays

The inhibitory activity of coumarins **4**–**45** was investigated through a stopped-flow CO_2_ hydrase assay [23] using acetazolamide (AAZ) as reference compound. Compounds **4**–**45** were tested against the human cancer-related isoforms CA IX and XII, considering the cytosolic isoforms *h*-CA I and *h*-CA II as off-target isoforms. A structure activity relationship has been determined for each series, considering the inhibition constants (K_i_) reported in Table 1, Table 2 and Table 3.

All of the benzylamides **4**–**25** (Table 1) showed no inhibition on off-target isoforms *h*-CA I and II, proving that 3-substituted 7-hydroxycoumarin is a privileged scaffold in the development of selective *h*-CA IX/XII inhibitors. Moreover, several compounds of the series displayed different selectivity towards *h*-CA IX or *h*-CA XII, while only compound **25,** endowed with a naphthyl moiety, showed K_i_ in the low micromolar range for both *h*-CA IX (K_i_ = 18.3 µM) and *h*-CA XII (K_i_ = 9.6 µM). The substitution of the naphthyl ring with a phenyl ring (compound **4**) was related to a reduction of activity against *h*-CA IX (K_i_ = 42.2 µM), while the activity on *h*-CA XII was maintained (K_i_ = 7.5 µM). It is interesting to see how the inhibitory activities changed when a methyl group was introduced in the 2-, 3-, or 4-position of the phenyl ring. The 2-methylbenzyl-substituted compound **5** showed a slightly better activity on *h*-CA IX (K_i_ = 25.4 µM) compared with *h*-CA XII (K_i_ = 35.5 µM). The shift of the methyl group into the 3-position (compound **6**) or 4-position (compound **7**) improved selectivity on *h*-CA XII (compound **6** K_i_ = 9.3 µM, compound **7** K_i_ = 8.8 µM) if compared with *h*-CA IX (compound **6** K_i_ = 82.5 µM, compound **7** K_i_ = 33.7 µM). The introduction of a methoxy group on the phenyl ring was related to a decrease of inhibitory activity against both *h*-CA IX and XII, with the only exception for compound **9**, endowed with a 4-methoxy group in which the activity on *h*-CA XII was recovered (K_i_ = 9.1 µM). The introduction of a nitro group in both the 3- (compound **12**) and 4-position (compound **11**) reduced the inhibitory activity on cancer-related isoforms and the same trend was observed by introducing a fluorine atom (compounds **13** and **14**) or a trifluoromethyl group (compounds **15**, **16** and **17**). On the other hand, the presence of one or two chlorine atoms strongly influenced the inhibitory activity. The 2-chlorobenzyl derivative **19** was found to be about three times more active on *h*-CA IX (K_i_ = 19.1 µM) compared with the activity on *h*-CA XII (K_i_ = 64.9 µM). Moving the chlorine atom in 3-position (compound **20**) resulted in a decrease of the inhibitory activity on *h*-CA IX (K_i_ = 33.9 µM) while the activity on *h*-CA XII is maintained (K_i_ = 42.6 µM). Furthermore, an inversion of the selectivity moving the chlorine atom into 4-position was observed (compound **21**, K_i_ = 80.7 µM on *h*-CA IX and K_i_ = 39.7 µM on *h*-CA XII). The *h*-CA IX selectivity was recovered by adding a second chlorine atom in 2-position (compound **22**, K_i_ = 22.0 µM on *h*-CA IX and K_i_ = 84.1 µM on *h*-CA XII), while, in the 3,4-dichlorophenyl analogue, an inversion of the selectivity was achieved again (compound **23**, K_i_ = 46.2 µM on *h*-CA IX and K_i_ = 21.0 µM on *h*-CA XII). Surprisingly, the introduction of a second chlorine atom to give the 2,6-dichlorobenzyl analogue **24** was not tolerated (K_i_ = 85.6 µM on *h*-CA IX and K_i_ = 75.1 µM on *h*-CA XII).

Compounds **26**–**30** (Table 2) are characterized by the presence of an additional methylene group between the coumarinamide core and the aryl moiety, and, luckily, even in this case all of the compounds were found to to be inactive on the off-target isoforms *h*-CA I and II. Most of these were found to be active on the cancer-related isoforms *h*-CA IX and XII at high micromolar levels with the exception of compounds **29** and **30**. These two compounds are characterized by the presence of 2-chlorine (compound **29**) and 3-chlorine atoms (compound **30**), displayed activity at low micromolar range (compound **29** K_i_ = 5.5 µM on *h*-CA IX, compound **30** K_i_ = 7.5 µM on *h*-CA IX) and showed a good selectivity against *h*-CA IX over *h*-CA XII. The introduction on the phenyl ring of a fluorine atom decreased the inhibitory activity as observed for compound **28**, as well as the presence of one or two methoxy groups (compounds **26** and **27**, respectively).

Furthermore, we introduced a piperazine ring as a spacer between the coumarin core and the substituted aromatic ring, in order to evaluate the modifications of the inhibitory activity in presence of a more rigid spacer. Even in this case, amides **31**–**45** (Table 3) were found to be inactive on *h*-CA I and II, confirming the great selectivity of this new class of substituted 7-hydroxy coumarins. Compound **31,** endowed with a benzyl ring bound to piperazine, showed an activity in high micromolar range on both *h*-CA IX and XII, with a small selectivity on *h*-CA XII (K_i_ = 84.1 µM on *h*-CA IX, K_i_ = 59.5 µM on *h*-CA XII). The substitution of the benzyl ring with the phenyl ring (compound **32**) slightly improved the activity on *h*-CA IX (K_i_ = 49.3 µM), but most importantly highly improved the activity on *h*-CA XII, reaching the low micromolar range (K_i_ = 9.1 µM) and about a 5-fold selectivity on *h*-CA XII over *h*-CA IX. The introduction of a methyl group on the 3- or 4-position of the aryl ring decreased the inhibitory activity on both cancer-related isoforms (compound **33** K_i_ = 89.3 µM on *h*-CA IX, K_i_ = 40.5 µM on h-CA XII; compound **34** K_i_ = 83.2 µM on *h*-CA IX, K_i_ = 56.0 µM on *h*-CA XII). The introduction of a second methyl group in 2-position (compounds **35**–**37**) did not improve the inhibitory activity, while the presence of a 3,4-dimethylphenyl moiety (compound **38**) selectively increased the activity only on *h*-CA XII (K_i_ = 9.2 µM).

On the other hand, the 3,5-dimethylphenyl analog **39** displayed the worst activity on *h*-CA XII (K_i_ = 46.8 µM). The introduction of a trifluoromethyl group to give compound **45** improved the activity on both *h*-CA XII (K_i_ = 8.4 µM) and *h*-CA IX *(*K_i_ = 22.6 µM). The activity on *h*-CA IX was retained by substitution of the trifluoromethyl group with nitro group, while the activity on *h*-CA XII decreased (compound **42** K_i_ = 30.1 µM on CA IX, K_i_ = 62.8 µM on *h*-CA XII). Moving the nitro group into 3-position did not affect the activity on *h*-CA IX, while the activity on *h*-CA XII was slightly improved (compound **41** K_i_ = 25.4 µM on *h*-CA IX, K_i_ = 47.5 µM on *h*-CA XII). Furthermore, the presence of 2-nitrophenyl was not tolerated (compound **40** K_i_ = 85.2 µM on *h*-CA IX, K_i_ = 63.8 µM on *h*-CA XII). Moreover, the introduction of a methyl group in 4-position generated compound **44*,*** which showed activity at low micromolar range against both *h*-CA IX and *h*-CA XII (K_i_ = 32.8 µM on *h*-CA IX, K_i_ = 22.5 µM on *h*-CA XII) while the 3-methoxyphenyl analogue **43** maintained about the same activity only on *h*-CA IX (K_i_ = 40.4 µM on *h*-CA IX, K_i_ = 79.9 µM on *h*-CA XII).

### 2.3. Docking Studies

**Scheme 2:**
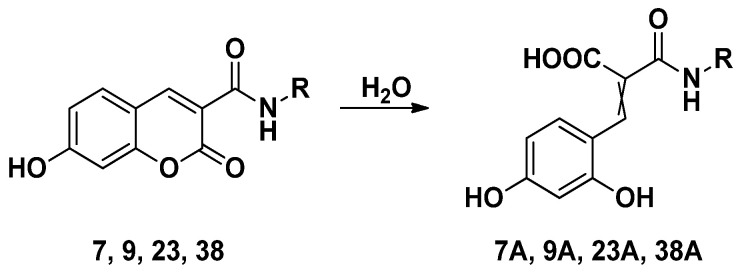
Hydrolysis of coumarins **7**, **9**, **23** and **38** to 2,4-dihydroxycinnamic acid amides **7A**, **9A**, **23A** and **38A**.

Molecular docking was performed to investigate the binding modes of the most promising CA inhibitors—**7**, **9**, **23** and **38**, which proved to be effective also on cell-based studies—into *h*-CA IX and *h*-CA XII isoforms. It is widely recognized that coumarins act as a prodrug of CA as they are subjected to the esterase activity of the enzyme leading to the hydrolysis of the lactone ring into the corresponding *E/Z* hydroxycinnamic derivatives [24,25]. According to crystallographic studies performed on *h*-CA II, coumarin hydrolysis provides the hydroxycinnamic acid in the less stable *Z* configuration being stabilized by the interaction with the enzyme, as observed for the natural coumarin 6-(1*S*-hydroxy-3-methylbutyl)-7-methoxy-2*H*-chromen-2-one [25]. Instead, small ligands, such as the unsubstituted 2-hydroxycinnamic acid, could undergo cis-trans isomerization within the CA active site interacting with the enzyme in their *E* configuration [26]. Therefore, based on the size of the analyzed derivatives, it is reasonable to hypothesize that the *Z* isomer could be responsible for the observed inhibitory activity (Scheme 2). However, as no experimental confirmation is available, docking calculations were focused on the hydrolyzed forms of the above cited *h*-CA IX and *h*-CA XII inhibitors as both *Z* and *E* isomers.

**Figure 2:**
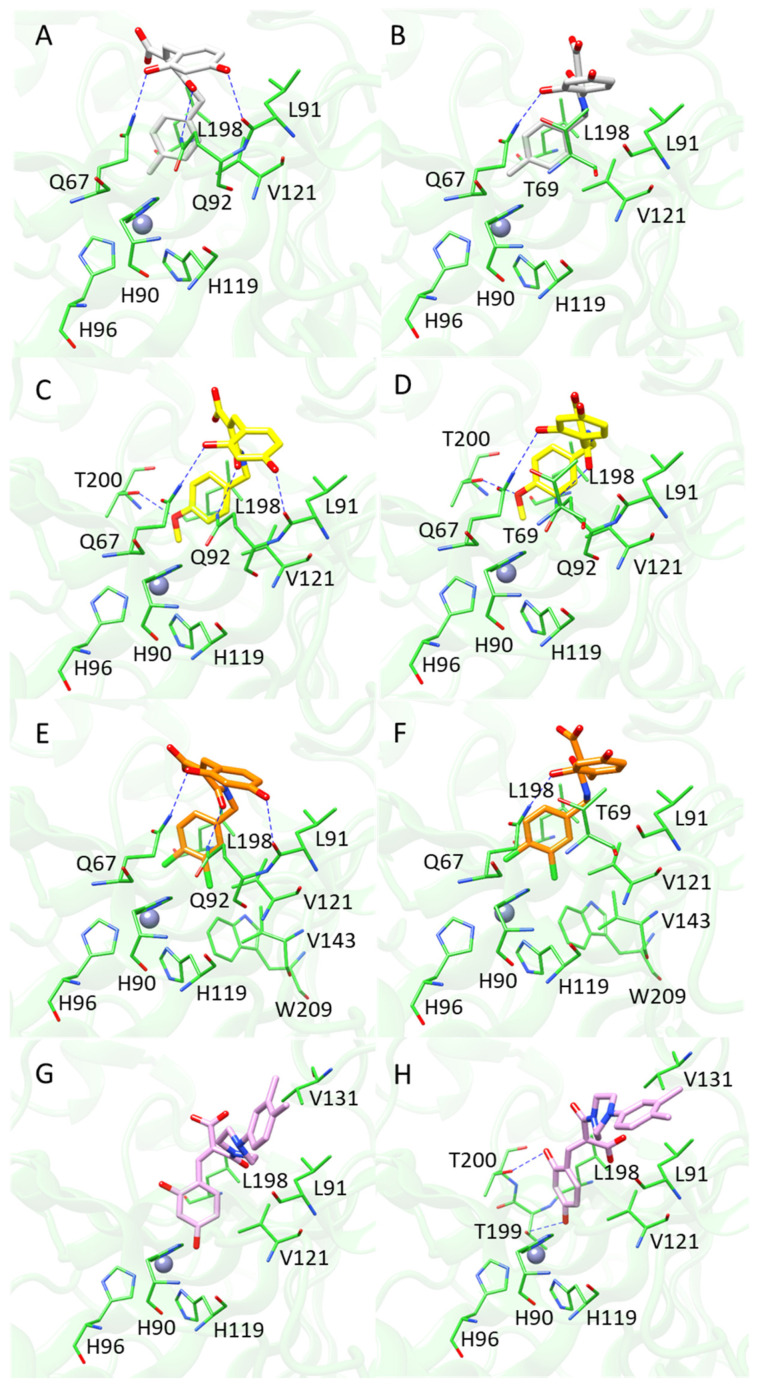
Docking poses of the *E/Z* isomers of hydrolyzed coumarins **7A**, **9A**, **23A** and **38A** into *h*CA IX. (**A**) (*E*)-**7A**, (**B**) (*Z*)-**7A**, (**C**) (*E*)-**9A**, (**D**) (*Z*)-**9A**, (**E**) (*E*)-**23A**, (**F**) (*Z*)-**23A**, (**G**) (*E*)-**38A**, (**H**) (Z)-**38A**.

When bound to *h*-CA IX, the hydrolyzed benzylamide derivatives **7A**, **9A** and **23A** displayed quite similar binding modes in both their *E* (Figure 2A,C,E) and *Z* configurations (Figure 2B,D,F). In more detail, in both isomers, the benzyl ring occupies a region lined by H90, V121 and L198 which is implicated in hydrophobic contact with the inhibitors. Two slightly different orientations were observed for the resorcinol ring derived from the hydrolysis of the coumarin system. In both isomers, this moiety is located in proximity of L91, establishing hydrophobic interactions with its side chain and H bond with Q67 through the hydroxyl group at the ortho position. Instead, the hydroxyl group at para position points towards the backbone of L91 in the *E* isomers engaging in H bonds with its carbonyl group, while it is oriented in proximity of T69 in the *Z* isomers. Furthermore, the carbonyl group of all *E* isomers and the (*Z*)-hydrolyzed coumarin **7A** form an H bond with Q92. An additional H bond was observed between the methoxy group of (*E*)-**9A** and the side chain of T200 (Figure 2C), while in the case of **23A** the presence of the chlorine atoms favors the formation of further hydrophobic interactions with V143 and W209 (Figure 2E,F). The *E/Z* isomers of the hydrolyzed phenylpiperazine **38A** adopt different orientations if compared with the benzylamide analogs. In this case the resorcinol moiety is positioned in proximity to V121 and L198, establishing hydrophobic contacts, while the phenylpiperazine moiety occupies a hydrophobic niche lined by V131, L91 and V121, which engage in hydrophobic interactions with their side chains (Figure 2G,H). H bonds were observed between the hydroxyl groups of the *Z* isomer and the side chains of T199 and T200. No other relevant interactions were detected for the *E* isomer which can explain the lower inhibitory activity towards *h*-CA IX observed for compound **38A** in respect to the previously analyzed benzylamide derivatives.

**Figure 3:**
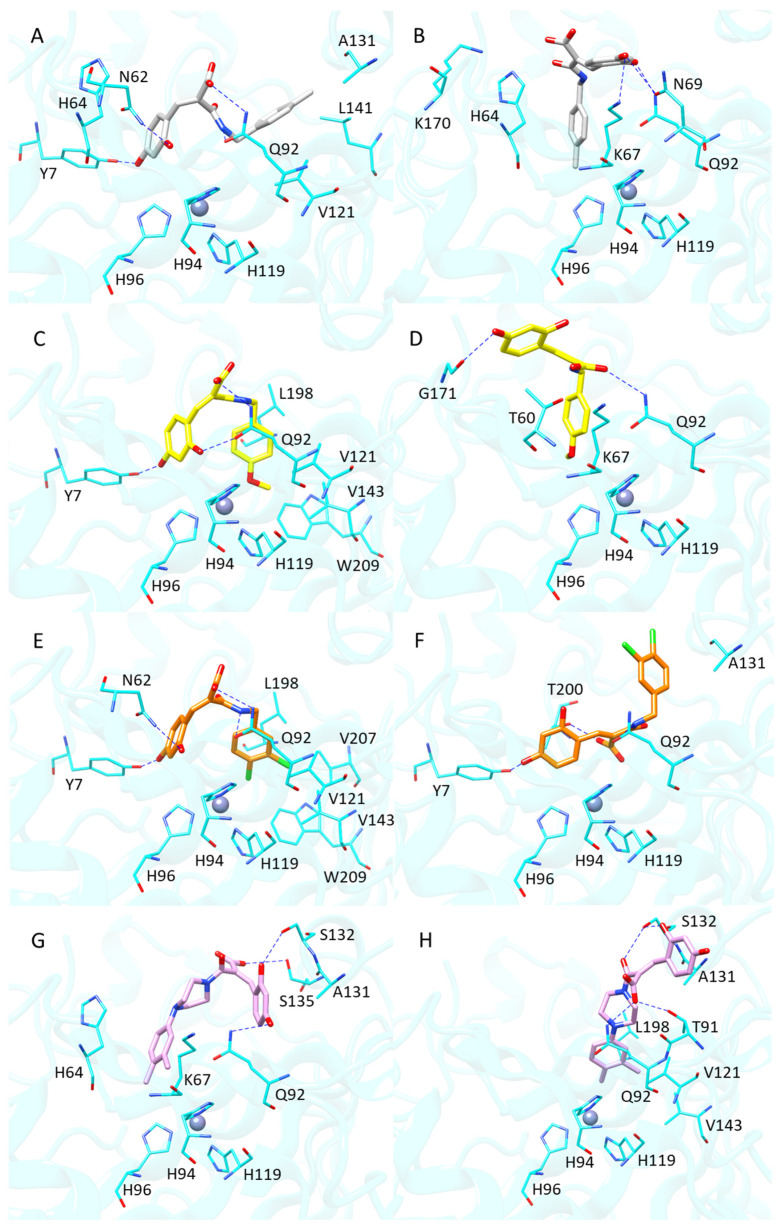
Docking poses of the *E/Z* isomers of hydrolyzed coumarins **7A**, **9A**, **23A** and **38A** into *h*-CA XII. (**A**) (*E*)-**7A**, (**B**) (*Z*)-**7A**, (**C**) (*E*)-**9A**, (**D**) (*Z*)-**9A**, (**E**) (*E*)-**23A**, (**F**) (*Z*)-**23A**, (**G**) (*E*)-**38A**, (**H**) (*Z*)-**38A**.

Docking results with regard to *h*-CA XII revealed that the resorcinol ring of the hydrolyzed coumarins **7A**, **9A** and **23A** in their *E* configurations is oriented towards Y7 engaging in an H bond with its side chain through the hydroxyl group at para position. Instead, the hydroxyl group at the ortho position is involved in an H bond with N62 in the case of (*E*)-**7A** (Figure 3A) and (*E*)-**23A** (Figure 3C), and with Q92 in the case of (*E*)-**7A** (Figure 3C). All *E* isomers of the benzylamide derivatives established a network of H bonds involving the amide group of Q92 and their carboxyl and amide groups. Regarding the hydrolyzed coumarin (*E*)-**7A**, the benzyl moiety occupy a sub-pocket lined by A131, L141 and V121, eliciting hydrophobic contacts (Figure 3A), while, in the docking poses of (*E*)-**7A** and (*E*)-**23A,** the same portion is inserted in a lipophilic niche lined by L198, V121, V143, W209 and H119, which establish hydrophobic interactions with their side chains. Moreover, aromatic interactions were observed between i) the resorcinol moiety of (*E*)-**7A** and H64 and H94, and ii) both the resorcinol and the benzyl rings of (*E*)-**9A** and H94. These additional interactions might contribute to the higher affinity towards hCA XII observed for coumarins **7** and **9** if compared with **23**. Concerning the *Z* isomers, the hydrolyzed coumarins **7A** (Figure 3B) and **9A** (Figure 3D) established hydrophobic contacts with H96 through their methyl and methoxy groups, respectively, and aromatic interactions with H94 through the benzyl moiety. Moreover, this portion is involved in further aromatic interactions with H64 in (*Z*)-**7A** and in additional hydrophobic contacts with T60 in the case of (*Z*)-**9A**. Instead, the *Z* isomer of the hydrolyzed coumarin **23A** (Figure 3F) orients the benzyl moiety in proximity of A131, which is involved in hydrophobic contacts with the inhibitor. The resorcinol moiety occupies different regions of the binding site in all analyzed *Z* forms of the benzylamide derivatives. In the docking pose of (*Z*)-**7A**, the resorcinol ring establishes a network of H bonds with K67, N69 and Q92, while it faces the opposite side in (*E*)-**9A,** engaging H bonds with G171. In the case of (*Z*)-**23A** the resorcinol ring is involved in H bonds with Y7 and aromatic interactions with H94. Additional H bonds were observed between the carboxyl group of i) (*Z*)-**9A** and Q92 and ii) (*Z*)-**23A** and T200. Interestingly, in the binding modes obtained for (*Z*)-**7A** and (*Z*)-**9A,** the carboxyl group forms a salt bridge with the peculiar K170 and K67, respectively, which might be responsible for the higher affinity observed for coumarins **7** and **9** with respect to **23**. Finally, regarding the hydrolyzed forms of the phenylpiperazine derivative **38A**, the results show that the *E* isomer binds to *h*CA XII, with the resorcinol ring establishing H bonds with S132 and Q92 and hydrophobic contacts with A131 (Figure 3G). The carboxyl group is engaged in H bonds with S135 while the benzyl moiety establishes pi-stacking interactions with H64 and hydrophobic contacts with H94, H96 and K67. Instead, in the *Z* isomer the benzyl portion occupies a sub-pocket lined by H94, L198, V121 and V143, eliciting hydrophobic contacts with the surrounding residues (Figure 3H). The carboxyl group is involved in H bonds with Q92 and T91, while the carbonyl group and the hydroxyl group at the ortho position of the resorcinol ring form H bonds with S132, which is involved in H bonds with Q92 and T91. Meanwhile the carboxyl group and the hydroxyl group at the ortho position of the resorcinol ring form H bonds with S132.

Docking studies have highlighted that the inhibition mechanism of the analyzed coumarin derivatives could be related to the ability of their hydrolyzed products to interact with the residues located at the entrance of *h*-CA IX and *h*-CA XII active sites, in agreement with literature data [25,26]. This region of pocket presents the highest variability in terms of amino acid composition among the different CA isoforms. Therefore, the peculiar binding mode of coumarins enables to tune the selectivity of this class of inhibitors towards specific *h*-CA isoforms, allowing the development of highly isoform-selective *h*-CA inhibitors. In addition, docking outcomes suggested that the major selectivity of the selected compounds towards *h*-CA XII might be due to their capability to interact with non-conserved residues of *h*-CA XII binding sites, including S132 (D132 in *h*-CA IX), S135 (L135 in *h*-CA IX), K170 (E170 in *h*-CA IX), N69 (T69 in *h*-CA IX) and K67 (Q67 in *h*-CA IX), which allows for the instauration of peculiar H bonds and/or salt bridge with the ligands, except for coumarin **23**. Indeed, in both of its *E/Z* hydrolyzed forms, compound **23** exhibited an interaction pattern involving residues that are conserved among *h*-CA IX and *h*-CA XII isoforms, which can explain its reduced selectivity towards *h*-CA XII if compared with the other analyzed derivatives.

### 2.4. Biological Effects of Coumarin Amides

**Figure 4:**
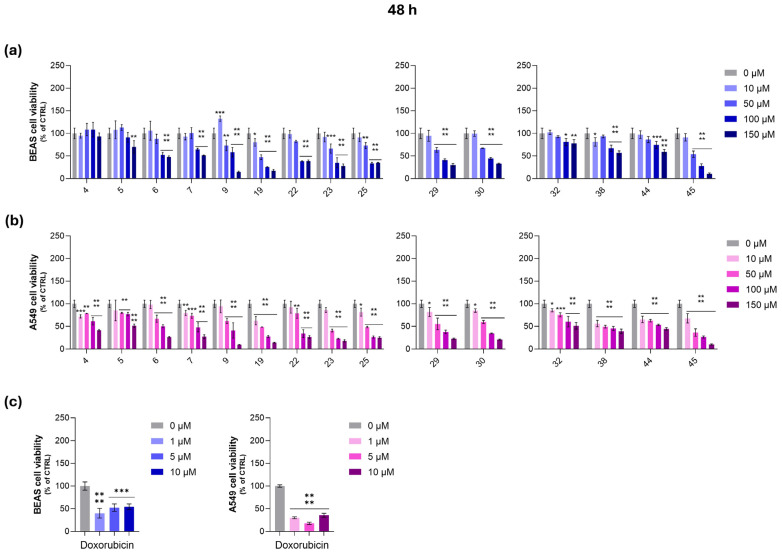
Cell viability of BEAS (**a**) and (**b**) A549 cells exposed to increasing concentrations (0–150 µM) of selected compounds (**4**-**7**, **9**, **19**, **22**, **23**, **25**, **29**, **30**, **32**, **38**, **44** and **45**) for 48 h. (**c**) Cell viability of BEAS and A549 cells exposed to doxorubicin (0-10 µM) after 48 h of exposure. Bar graphs represent cell viability percentages. The untreated control (CTRL = 0 µM) is set as the 100%. Data are presented as means ± standard deviations obtained from one experiment in triplicates (n = 3). * = *p* < 0.01, ** = *p* < 0.001, *** = *p* < 0.0001, **** = *p* < 0.00001 comparing treated to the untreated control.

Coumarin amides showing high inhibitory activity against *h*-CA IX and *h*-CA XII were tested for their biological effects on bronchial adenocarcinoma cells (A549) and on normal bronchial epithelial cells (BEAS-2B) to assess selectivity. (Figure 4). Firstly, cell viability was determined by MTT assay at 24 and 48 h exposure time. The best exposure time was found to be 48 h, demonstrating that these compounds are weakly effective after a 24 h exposure (Supplementary Information, Figure S1). In parallel, doxorubicin was administered as comparison on both the cell lines (Figure 4c). As shown, both BEAS and A549 cell viability is dramatically decreased by doxorubicin already at 1 µM, thus highlighting the absence of selectivity.

Based on the MTT data shown in Figure 4, a selectivity index was determined by calculating the IC_50_-BEAS/IC_50_ A549 ratio for each compound. Compound **38** was found to be the best compound of the series, as its selectivity index was assessed at 3.747 and thus achieving the highest ratio. Furthermore, the IC_50_ on A549 cells was calculated in the medium–low micromolar range (50.02 µM). The compounds **7**, **9** and **23** that are able to decrease malignant cell viability (IC_50_ on A549 cells 85.78, 68.20 and 39.50 µM, respectively) and which have a selectivity index higher than 1.5 (Table 4) were selected for further analyses.

**Figure 5:**
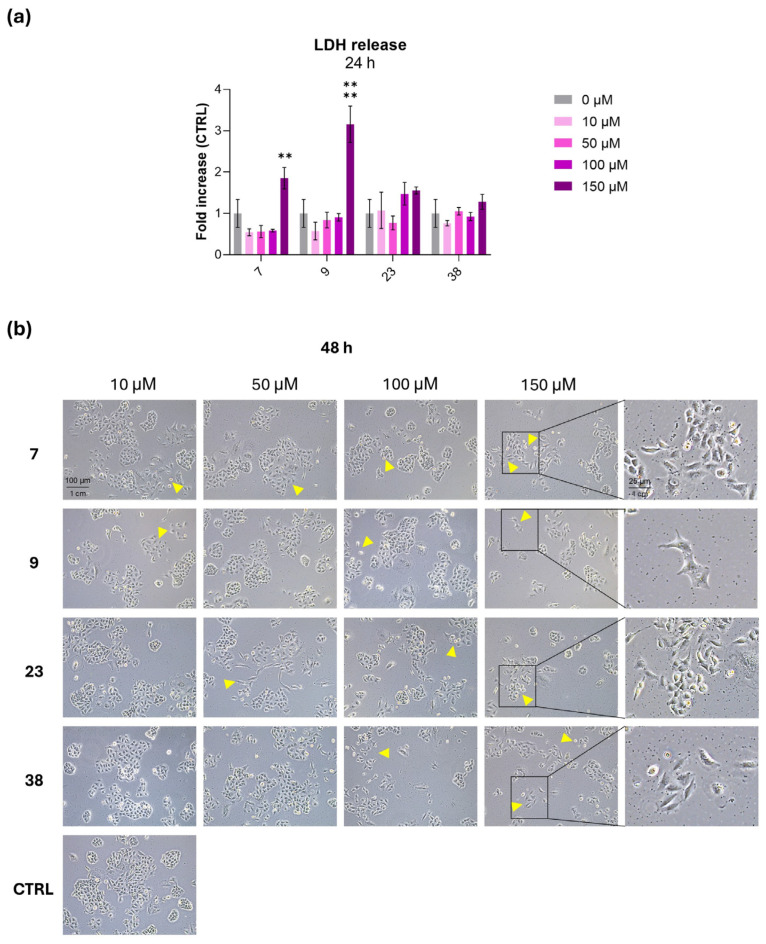
(**a**) Cytotoxicity occurrence in A549 cells exposed to increasing concentrations (0–150 µM) of compounds **7**, **9**, **23** and **38** after 24 h. The bar graphs show the amount of lactate dehydrogenase (LDH) released from treated A549 cells as a fold increase respective to that secreted by untreated cells (CTRL = 0 µM) after 24 h of exposure. (**b**) Light phase-contrast images of A549 cells 48 h after treatment. Magnification 10×. Yellow arrows highlight morphological changes in the cell population. Data are presented as means ± standard deviations obtained from one experiment in triplicates (n = 3). ** = *p* < 0.001, **** = *p* < 0.00001 comparing treated to the untreated control.

To assess cytotoxicity occurrence, A549 cells were afterwards exposed to the selected compounds **7, 9**, **23** and **38** for 24 h and the release of lactic dehydrogenase (LDH) was quantified in cell supernatants. None of the compounds administered displayed cytotoxicity, except for **7** and **9** at the highest concentration administered (150 µM). Specifically, the amount of LDH was found to be lower than when released by the untreated control or not significantly higher (Figure 5a). In parallel, when compounds **7** and **9** were used at 150 µM, the fold increase rose to almost 2 for compound **7** and at almost 3 for compound **9**.

The observation at the phase-contrast microscope revealed that there was a decrease in the number of cell clusters, making it plausible to assume that the decrease of cell viability assessed through the MTT assay is consistent with a decrease of cell proliferation rather than cytotoxicity occurrence. Moreover, a more detailed observation highlighted that a change in cell morphology can be assessed (Figure 5b).

**Figure 6:**
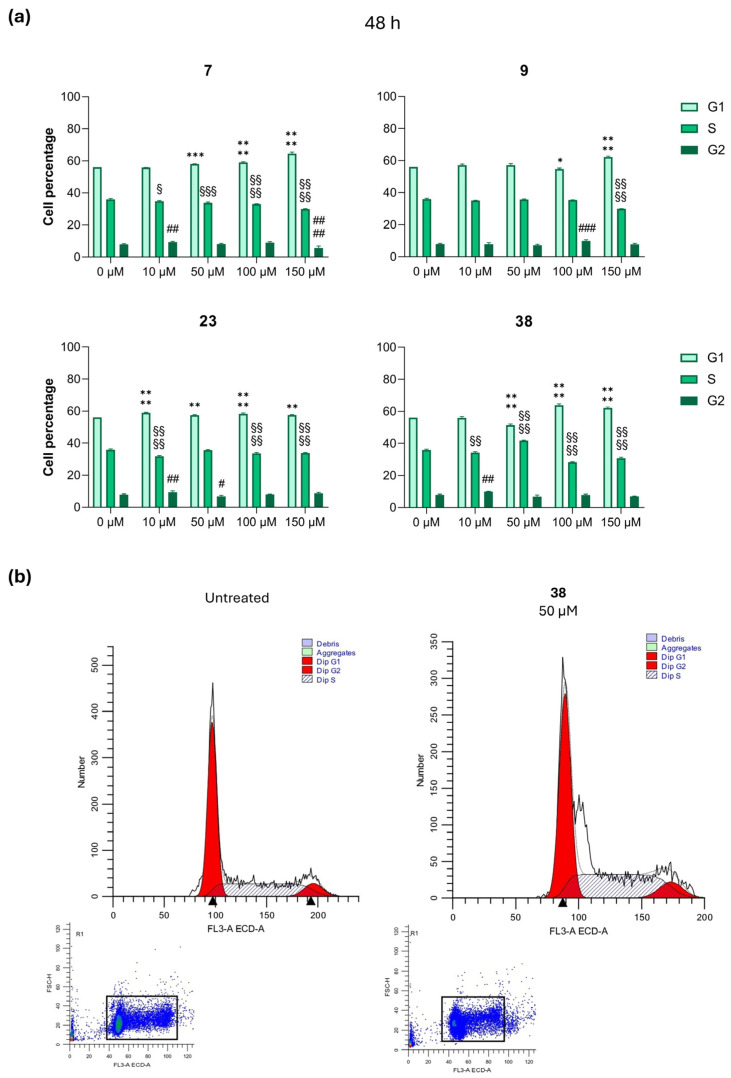
(**a**) Cell cycle analysis in A549 cells exposed to increasing concentrations (0–150 µM) of compounds **7**, **9**, **23** and **38** after 48 h. Data are presented as means ± standard deviations from three independent experiments (n = 3). Bars highlight cell percentages in the various phases of the cell cycle (G1, S, and G2) of A549. * = *p* < 0.01, ** = *p* < 0.001, *** = *p* < 0.0001, **** = *p* < 0.00001 comparing treated with the untreated control (G1 phase). § = *p* < 0.01, §§ = *p* < 0.001, §§§ = *p* < 0.0001, §§§§ = *p* < 0.00001 comparing treated with the untreated control (S phase). # = *p* < 0.01, ## = *p* < 0.001, ### = *p* < 0.0001, #### = *p* < 0.00001 comparing treated with the untreated control (G2 phase). (**b**) The top panel displays the DNA profile of cells 48 h after treatment. Peaks are generated by the emission of PI in the FL3 fluorescence channel. The bottom panel is a dot plot representing the gating strategy.

Treated cells are more elongated compared with those in the untreated sample, which display a typical epithelial morphology, with several clusters containing hexagonal or irregular scale-like shapes. Notably, images highlight a dose-dependent decrease of cell clusters, independently from the compound, thus reflecting lower proliferation Because of these observations, an analysis of the cell cycle was performed to assess that an arrest of cell proliferation might be caused by selected compounds (Figure 6 and Figure S2).

The analysis of the cell cycle revealed **23** and **38** as the best compounds of the series in terms of the arrest of cell cycle progression (Figure 6a). These compounds were able to arrest cells at the G1/S checkpoint, being percentages of cells found in the S phase that are significantly increased when compared with the untreated control.

## 3. Materials and Methods

### 3.1. Chemistry

All commercially available solvents and reagents were used without further purification. NMR spectra were recorded on a Bruker Advance III HD 600 MHz (Bruker, Bremen, Germany). The chemical shifts (*δ*) are reported in part per million downfield from tetramethylsilane (TMS), which was used as internal standard. The spectra were recorded in hexadeuteriodimethylsulphoxide (DMSO-*d*_6_). Infrared spectra were recorded on a Nicolet iS10 spectrometer (Thermo Fisher Scientific Inc, Paisley, UK). The main bands are given in cm^−1^. Positive-ion electrospray ionization (ESI) mass spectra were recorded on a double-focusing MAT 95 instrument (Finnigan, Waltham, MA, USA) with BE geometry. Melting points (mp) were determined with an SMP1 Melting Point apparatus (Stuart Scientific, Stone, UK) and are uncorrected. All products reported showed ^1^H NMR spectra in agreement with the assigned structures. The purity of the tested compounds was determined by combustion elemental analyses conducted by the Microanalytical Laboratory of the Chemistry Department of the University of Ferrara with a MT-5 CHN recorder elemental analyzer (Yanagimoto, Kyoto, Japan) and the values found were within 0.4% of theoretical values. 7-Hydroxy-2-oxo-2*H*-chromene-3-carboxylic acid **3** was prepared as previously described [21].

#### General Procedure for the Preparation of 7-Hydroxy-2-Oxo-2*H*-Chromene-3-Carboxamides (**4**–**45**)

7-Hydroxy-2-oxo-2*H*-chromene-3-carboxylic acid **3** (0.21 g, 1 mmol), EDCI (0.19 g, 1 mmol) and HOBt (0.13 g, 1 mmol) were dissolved in anhydrous MeCN (5 mL). The resulting mixture was stirred at r.t. for 30 min, then the substituted amine (1 mmol) was added. The mixture was stirred at r.t. for 6 h. The solvent was removed under vacuum and the residue was dissolved in ethyl acetate (10 mL) and washed sequentially with water (2 × 15 mL), saturated NaHCO_3_ aqueous solution (2 × 15 mL), 10% citric acid (2 × 15 mL) and brine (2 × 10 mL). The organic layer was dried over anhydrous sodium sulfate (Na_2_SO_4_), filtered and evaporated under reduced pressure. The residues were treated with diethyl ether (Et_2_O) and the formed solids were filtered off and recrystallized from MeOH to give the corresponding amides **4**–**45**.

##### *N-Benzyl-7-Hydroxy-2-Oxo-2H-Chromene-3-Carboxamide* **(4)** [27]

Following the general procedure, the title compound was prepared starting from benzylamine. Yield 85% M.p. 166–167 °C. ^1^H NMR (DMSO-*d*_6_) δ 4.53 (d, *J* = 5.9 Hz, 2H, CH_2_); 6.77 (d, *J* = 2.1 Hz, 1H, Ar); 6.85 (dd, *J* = 8.6 2.2 Hz, 1H, Ar); 7.26 (m, 2H, Ar); 7.33 (m, 3H, Ar); 7.79 (d, *J* = 8.5 Hz, 1H, Ar); 8.79 (s, 1H, Ar); 9.05 (t, *J* = 5.9 Hz, 1H, NH); 11.11 (s, 1H, OH). IR 3374, 1782, 1727 cm^−1^. Elemental analysis: calculated for C_17_H_13_NO_4_ (295.29)%C 69.15, %H 4.44, %N 4.74, found %C 69.14 %H 4.43, %N 4.73. *m*/*z* 296.

##### *7-Hydroxy-N-(2-Methylbenzyl)-2-Oxo-2H-Chromene-3-Carboxamide* **(5)**

Following the general procedure, the title compound was prepared starting from 2-methylbenzylamine. Yield 67% M.p. 184–185 °C. ^1^H NMR (DMSO-*d*_6_) δ 2.31 (s, 3H, CH_3_); 4.51 (d, *J* = 5.8 Hz, 2H, CH_2_); 6.80 (d, *J* = 1.9 Hz, 1H, Ar); 6.88 (dd, *J* = 8.7 2.2 Hz, 1H, Ar); 7.17 (m, 3H, Ar); 7.26 (d, *J* = 8.1 Hz, 1H, Ar); 7.82 (d, *J* = 8.5 Hz, 1H, Ar); 8.80 (s, 1H, Ar); 8.96 (t, *J* = 5.7 Hz, 1H, NH); 11.10 (s, 1H, OH). IR 3362, 1784, 1729 cm^−1^. Elemental analysis: calculated for C_18_H_15_NO_4_ (309.32)%C 69.89, %H 4.89, %N 4.53, found %C 69.88 %H 4.87, %N 4.54. *m*/*z* 310.

##### *7-Hydroxy-N-(3-Methylbenzyl)-2-Oxo-2H-Chromene-3-Carboxamide* **(6)**

Following the general procedure, the title compound was prepared starting from 3-methylbenzylamine. Yield 59% M.p. 194–195 °C. ^1^H NMR (DMSO-*d*_6_) δ 2.28 (s, 3H, CH_3_); 4.49 (d, *J* = 5.8 Hz, 2H, CH_2_); 6.80 (d, *J* = 1.5 Hz, 1H, Ar); 6.88 (dd, *J* = 8.6 1.9 Hz, 1H, Ar); 7.06 (d, *J* = 7.3 Hz, 1H, Ar); 7.13 (m, 2H, Ar); 7.21 (t, *J* = 7.5 Hz, 1H, Ar); 7.81 (d, *J* = 8.5 Hz, 1H, Ar); 8.80 (s, 1H, Ar); 9.01 (t, *J* = 5.6 Hz, 1H, –NH); 11.13 (s, 1H, –OH). IR 3363, 1784, 1730 cm^−1^. Elemental analysis: calculated for C_18_H_15_NO_4_ (309.32)%C 69.89, %H 4.89, %N 4.53, found %C 69.90 %H 4.87, %N 4.53. *m*/*z* 310.

##### *7-Hydroxy-N-(4-Methylbenzyl)-2-Oxo-2H-Chromene-3-Carboxamide* **(7)** [27]

Following the general procedure, the title compound was prepared starting from 4-methylbenzylamine. Yield 71% M.p. 190–191 °C. ^1^H NMR (DMSO-*d*_6_) δ 2.28 (s, 3H, CH_3_); 4.48 (d, *J* = 5.9 Hz, 2H, CH_2_); 6.81 (d, *J* = 1.9 Hz, 1H, Ar); 6.86 (dd, *J* = 8.6 2.1 Hz, 1H, Ar); 7.14 (d, *J* = 7.8 Hz, 2H, Ar); 7.22 (d, *J* = 7.8 Hz, 2H, Ar); 7.81 (d, *J* = 8.8 Hz, 1H, Ar); 8.80 (s, 1H, Ar); 8.99 (t, *J* = 6.0 Hz, 1H, –NH); 11.21 (s, 1H, –OH). IR 3364, 1782, 1731 cm^−1^. Elemental analysis: calculated for C_18_H_15_NO_4_ (309.32)%C 69.89, %H 4.89, %N 4.53, found %C 69.89 %H 4.88, %N 4.53. *m*/*z* 310.

##### *7-Hydroxy-N-(2-Methoxybenzyl)-2-Oxo-2H-Chromene-3-Carboxamide* **(8)**

Following the general procedure, the title compound was prepared starting from 2-methoxylbenzylamine. Yield 58% M.p. 184–185 °C. ^1^H NMR (DMSO-d_6_) δ 3.84 (s, 3H, OCH_3_); 4.49 (d, *J* = 5.8 Hz, 2H, CH_2_); 6.81 (m, 1H, Ar); 6.90 (m, 2H, Ar); 7.01 (d, *J* = 7.8 Hz, 1H, Ar); 7.22 (d, *J* = 7.4 Hz, 1H, Ar); 7.26 (t, *J* = 7.8 Hz, 1H, Ar); 7.81 (d, *J* = 8.8 Hz, 1H, Ar); 8.79 (s, 1H, Ar); 9.05 (t, *J* = 5.9 Hz, 1H, NH); 11.10 (s, 1H, OH). IR 3390, 1775, 1728 cm^−1^. Elemental analysis: calculated for C_18_H_15_NO_5_ (325.32)%C 66.46, %H 4.65, %N 4.31, found %C 66.47 %H 4.65, %N 4.31. *m*/*z* 326.

##### *7-Hydroxy-N-(4-Methoxylbenzyl)-2-Oxo-2H-Chromene-3-Carboxamide* **(9)** [27]

Following the general procedure, the title compound was prepared starting from 4-methoxylbenzylamine. Yield 92% M.p. 188–189 °C. ^1^H NMR (DMSO-*d*_6_) δ 3.88 (s, 3H, OCH_3_); 4.42 (d, *J* = 5.3 Hz, 2H, CH_2_); 6.83 (m, 1H, Ar); 6.94 (d, *J* = 8.4 Hz, 1H, Ar); 7.08 (d, *J* = 8.1 Hz, 2H, Ar); 7.34 (d, *J* = 7.4 Hz, 2H, Ar); 7.83 (d, *J* = 8.6 Hz, 1H, Ar); 8.82 (s, 1H, Ar); 9.10 (t, *J* = 6.0 Hz, 1H, NH); 11.04 (s, 1H, OH). IR 3385, 1772, 1721 cm^−1^. Elemental analysis: calculated for C_18_H_15_NO_5_ (325.32)%C 66.46, %H 4.65, %N 4.31, found %C 66.47 %H 4.67, %N 4.30. *m*/*z* 326.

##### *7-Hydroxy-N-(3-Methoxybenzyl)-2-Oxo-2H-Chromene-3-Carboxamide* **(10)**

Following the general procedure, the title compound was prepared starting from 3-methoxylbenzylamine. Yield 64% M.p. 184–185 °C. ^1^H NMR (DMSO-*d*_6_) δ 3.86 (s, 3H, OCH_3_); 4.48 (d, *J* = 5.9 Hz, 2H, CH_2_); 6.78 (m, 1H, Ar); 6.94 (m, 2H, Ar); 7.12 (m, 1H, Ar); 7.26 (d, *J* = 7.2 Hz, 1H, Ar); 7.31 (t, *J* = 7.6 Hz, 1H, Ar); 7.89 (d, *J* = 8.5 Hz, 1H, Ar); 8.84 (s, 1H, Ar); 9.08 (t, *J* = 5.9 Hz, 1H, NH); 11.17 (s, 1H, OH). IR 3392, 1770, 1733 cm^−1^. Elemental analysis: calculated for C_18_H_15_NO_5_ (325.32)%C 66.46, %H 4.65, %N 4.31, found %C 66.46 %H 4.63, %N 4.32. *m*/*z* 326.

##### *7-Hydroxy-N-(4-Nitrobenzyl)-2-Oxo-2H-Chromene-3-Carboxamide* **(11)**

Following the general procedure, the title compound was prepared starting from 4-nitrobenzylamine. Yield 82% M.p. 177–178 °C. ^1^H NMR (DMSO-*d*_6_) δ 4.65 (d, *J* = 6.4 Hz, 2H, CH_2_); 6.80 (d, *J* = 2.0 Hz, 1H, Ar); 6.87 (dd, *J* = 8.6 2.2 Hz, 1H, Ar); 7.58 (d, *J* = 8.8 Hz, 2H, Ar); 7.80 (d, *J* = 8.6 Hz, 1H, Ar); 8.19 (d, *J* = 8.8 Hz, 2H, Ar); 8.79 (s, 1H, Ar); 9.23 (t, *J* = 6.1 Hz, 1H, NH); 11.10 (s, 1H, OH). IR 3385, 1772, 1721 cm^−1^. Elemental analysis: calculated for C_17_H_12_N_2_O_6_ (340.28)%C 60.00 %H 3.55, %N 8.23, found %C 60.00 %H 3.56, %N 8.23. *m*/*z* 341.

##### *7-Hydroxy-N-(3-Nitrobenzyl)-2-Oxo-2H-Chromene-3-Carboxamide* **(12)**

Following the general procedure, the title compound was prepared starting from 3-nitrobenzylamine. Yield 70% M.p. 171–172 °C. ^1^H NMR (DMSO-*d*_6_) δ 4.65 (d, *J* = 6.2 Hz, 2H, CH_2_); 6.88 (d, *J* = 2.3 Hz, 1H, Ar); 7.36 (m, 1H, Ar); 7.64 (m, 2H, Ar); 7.80 (d, *J* = 8.5 Hz, 1H, Ar); 8.11 (d, *J* = 8.6 Hz, 1H, Ar); 8.21 (m, 1H, Ar); 8.80 (s, 1H, Ar); 9.25 (t, *J* = 5.9 Hz, 1H, NH); 11.76 (s, 1H, OH). IR 3388, 1769, 1722 cm^−1^. Elemental analysis: calculated for C_17_H_12_N_2_O_6_ (340.28)%C 60.00 %H 3.55, %N 8.23, found %C 60.01 %H 3.57, %N 8.22. *m*/*z* 341.

##### *N-(4-Fluorobenzyl)-7-Hydroxy-2-Oxo-2H-Chromene-3-Carboxamide* **(13)** [27]

Following the general procedure, the title compound was prepared starting from 4-fluorobenzylamine. Yield 65% M.p. 180–181 °C. ^1^H NMR (DMSO-*d*_6_) δ 4.61 (d, *J* = 6.2 Hz, 2H, CH_2_); 6.81 (d, *J* = 2.2 Hz, 1H, Ar); 6.89 (dd, *J* = 8.4 2.3 Hz, 1H, Ar); 7.34 (d, *J* = 8.5 Hz, 2H, Ar); 7.55 (d, *J* = 8.1 Hz, 2H, Ar); 7.83 (d, *J* = 8.8 Hz, 1H, Ar); 8.84 (s, 1H, Ar); 9.15 (t, *J* = 5.4 Hz, 1H, NH); 11.08 (s, 1H, –OH). IR 3411, 1766, 1731 cm^−1^. Elemental analysis: calculated for C_17_H_12_FNO_4_ (313.28)%C 65.18, %H 3.86, %N 4.47, found %C 65.20 %H 3.86, %N 4.48. *m*/*z* 314.

##### *N-(3-Fluorobenzyl)-7-Hydroxy-2-Oxo-2H-Chromene-3-Carboxamide* **(14)**

Following the general procedure, the title compound was prepared starting from 3-fluorobenzylamine. Yield 77% M.p. 185–187 °C. ^1^H NMR (DMSO-*d*_6_) δ 4.63 (d, *J* = 6.4 Hz, 2H, CH_2_); 6.82 (m, 1H, Ar); 6.89 (d, *J* = 8.3 Hz,1H, Ar); 7.15 (m, 2H, Ar); 7.29 (d, *J* = 8.7 Hz, 1H, Ar); 7.67 (d, *J* = 8.5 Hz, 1H, Ar); 7.87 (d, *J* = 8.4 Hz, 1H, Ar); 8.80 (s, 1H, Ar); 9.11 (t, *J* = 5.5 Hz, 1H, NH); 11.11 (s, 1H, OH). IR 3432, 1762, 1726 cm^−1^. Elemental analysis: calculated for C_17_H_12_FNO_4_ (313.28)%C 65.18, %H 3.86, %N 4.47, found %C 65.18 %H 3.85, %N 4.49. *m*/*z* 314.

##### *7-Hydroxy-2-Oxo-N-(2-(Trifluoromethyl)benzyl)-2H-Chromene-3-Carboxamide* **(15)**

Following the general procedure, the title compound was prepared starting from 2-trifluoromethylbenzylamine. Yield 44% M.p. 189–190 °C. ^1^H NMR (DMSO-d_6_) δ 4.72 (d, *J* = 5.6 Hz, 2H, CH_2_); 6.81 (d, *J* = 1.7 Hz, 1H, Ar); 6.88 (dd, *J* = 8.6 2.1 Hz, 1H, Ar); 7.49 (t, *J* = 7.6, 1H, Ar); 7.56 (d, J = 7.6 Hz, 1H, Ar); 7.65 (t, *J* = 7.6 Hz, 1H, Ar); 7.73 (d, *J* = 7.8 Hz, 1H, Ar); 7.82 (d, *J* = 8.6 Hz, 1H, Ar); 8.80 (s, 1H, Ar); 9.15 (t, *J* = 5.9 Hz, 1H, NH); 11.23 (s, 1H, OH). IR 3364, 1782, 1731 cm^−1^. Elemental analysis: calculated for C_18_H_12_F_3_NO_4_ (363.29)%C 59.51, %H 3.33, %N 3.86, found %C 59.49 %H 3.34, %N 4.85. *m*/*z* 364.

##### *7-Hydroxy-2-Oxo-N-(3-(Trifluoromethyl)benzyl)-2H-Chromene-3-Carboxamide* **(16)**

Following the general procedure, the title compound was prepared starting from 3-trifluoromethylbenzylamine. Yield 81% M.p. 186–187 °C. ^1^H NMR (DMSO-d_6_) δ 4.61 (d, *J* = 6.2 Hz, 2H, CH_2_); 6.81 (d, *J* = 1.9 Hz, 1H, Ar); 6.88 (dd, *J* = 8.4 2.1 Hz, 1H, Ar); 7.57 (d, *J* = 8.3, 1H, Ar); 7.61 (d, *J* = 8.3 Hz, 1H, Ar); 7.65 (m, 1H, Ar); 7.70 (m, 1H, Ar); 7.81 (d, *J* = 8.5 Hz, 1H, Ar); 8.80 (s, 1H, Ar); 9.18 (t, *J* = 5.9 Hz, 1H, NH); 11.06 (s, 1H, OH). IR 3364, 1784, 1733 cm^−1^. Elemental analysis: calculated for C_18_H_12_F_3_NO_4_ (363.29)%C 59.51, %H 3.33, %N 3.86, found %C 59.50 %H 3.33, %N 4.87. *m*/*z* 364.

##### *7-Hydroxy-2-Oxo-N-(4-(Trifluoromethyl)benzyl)-2H-Chromene-3-Carboxamide* **(17)** [27]

Following the general procedure, the title compound was prepared starting from 3-trifluoromethylbenzylamine. Yield 67% M.p. 198–199 °C. ^1^H NMR (DMSO-d_6_) δ 4.66 (d, *J* = 6.5 Hz, 2H, CH_2_); 6.84 (m, 1H, Ar); 6.89 (d, *J* = 8.7 Hz, 1H, Ar); 7.64 (d, *J* = 8.8 Hz, 2H, Ar); 7.74 (d, *J* = 8.6 Hz, 2H, Ar); 7.85 (d, *J* = 8.3 Hz, 1H, Ar); 8.82 (s, 1H, Ar); 9.22 (t, *J* = 5.7 Hz, 1H, NH); 11.05 (s, 1H, OH). IR 3360, 1787, 1730 cm^−1^. Elemental analysis: calculated for C_18_H_12_F_3_NO_4_ (363.29)%C 59.51, %H 3.33, %N 3.86, found %C 59.52 %H 3.32, %N 4.86. *m*/*z* 364.

##### *N-(4-Bromobenzyl)-7-Hydroxy-2-Oxo-2H-Chromene-3-Carboxamide* **(18)**

Following the general procedure, the title compound was prepared starting from 4-bromobenzylamine. Yield 74% M.p. 195–196 °C. ^1^H NMR (DMSO-d_6_) δ 4.49 (d, *J* = 6.0 Hz, 2H, CH_2_); 6.79 (d, *J* = 1.8 Hz, 1H, Ar); 6.87 (dd, *J* = 8.6 2.0 Hz, 1H, Ar); 7.29 (d, *J* = 8.2 Hz, 2H, Ar); 7.52 (d, *J* = 8.2 Hz, 2H, Ar); 7.80 (d, *J* = 8.6 Hz, 1H, Ar); 8.78 (s, 1H, Ar); 9.09 (t, *J* = 5.9 Hz, 1H, NH); 11.12 (s, 1H, OH). IR 3431, 1762, 1726 cm^−1^. Elemental analysis: calculated for C_17_H_12_BrNO_4_ (374.19)%C 54.57, %H 3.23, %N 3.74, found %C 54.56, %H 3.21, %N 3.74. *m*/*z* 375.

##### *N-(2-Chlorobenzyl)-7-Hydroxy-2-Oxo-2H-Chromene-3-Carboxamide* **(19)**

Following the general procedure, the title compound was prepared starting from 2-chlorobenzylamine. Yield 72% M.p. 171–172 °C. ^1^H NMR (DMSO-*d*_6_) δ 4.52 (d, *J* = 5.3 Hz, 2H, CH_2_); 6.78 (m, 1H, Ar); 6.87 (d, *J* = 8.7 Hz, 1H, Ar); 7.15 (d, *J* = 7.6 Hz, 1H, Ar); 7.34 (m, 3H, Ar); 7.85 (d, *J* = 8.5 Hz, 1H, Ar); 8.82 (s, 1H, Ar); 9.15 (t, *J* = 5.7 Hz, 1H, NH); 11.08 (s, 1H, OH). IR 3384, 1775, 1727 cm^−1^. Elemental analysis: calculated for C_17_H_12_ClNO_4_ (329.73)%C 61.92, %H 3.67, %N 4.25, found %C 61.92 %H 3.68, %N 4.24. *m*/*z* 331.

##### *N-(3-Chlorobenzyl)-7-Hydroxy-2-Oxo-2H-Chromene-3-Carboxamide* **(20)**

Following the general procedure, the title compound was prepared starting from 3-chlorobenzylamine. Yield 68% M.p. 172–173 °C. ^1^H NMR (DMSO-*d*_6_) δ 4.53 (d, *J* = 5.8 Hz, 2H, CH_2_); 6.80 (m, 1H, Ar); 6.87 (d, *J* = 8.5 Hz, 1H, Ar); 7.34 (m, 4H, Ar); 7.80 (d, *J* = 8.7 Hz, 1H, Ar); 8.79 (s, 1H, Ar); 9.12 (t, *J* = 5.9 Hz, 1H, NH); 11.15 (s, 1H, OH). IR 3382, 1776, 1729 cm^−1^. Elemental analysis: calculated for C_17_H_12_ClNO_4_ (329.73)%C 61.92, %H 3.67, %N 4.25, found %C 61.91 %H 3.66, %N 4.25. *m*/*z* 331.

##### *N-(4-Chlorobenzyl)-7-Hydroxy-2-Oxo-2H-Chromene-3-Carboxamide* **(21)**

Following the general procedure, the title compound was prepared starting from 4-chlorobenzylamine. Yield 64% M.p. 180–181 °C. ^1^H NMR (DMSO-*d*_6_) δ 4.58 (d, *J* = 5.7 Hz, 2H, CH_2_); 6.82 (m, 1H, Ar); 6.89 (m, 1H, Ar); 7.18 (d, *J* = 7.9 Hz, 2H, Ar); 7.49 (d, *J* = 8.2 Hz, 2H, Ar); 7.89 (d, *J* = 8.8 Hz, 1H, Ar); 8.74 (s, 1H, Ar); 9.07 (t, *J* = 5.3 Hz, 1H, NH); 11.15 (s, 1H, OH). IR 3382, 1773, 1731 cm^−1^. Elemental analysis: calculated for C_17_H_12_ClNO_4_ (329.73)%C 61.92, %H 3.67, %N 4.25, found %C 61.92 %H 3.68, %N 4.27.*m*/*z* 331.

##### *N-(2,4-Dichlorobenzyl)-7-Hydroxy-2-Oxo-2H-Chromene-3-Carboxamide* **(22)**

Following the general procedure, the title compound was prepared starting from 2,4-dichlorobenzylamine. Yield 72% M.p. 177–178 °C. ^1^H NMR (DMSO-*d*_6_) δ 4.51 (d, *J* = 6.2 Hz, 2H, CH_2_); 6.80 (d, *J* = 2.1 Hz, 1H, Ar); 6.88 (dd, *J* = 8.5 2.2 Hz, 1H, Ar); 7.30 (dd, *J* = 8.3 2.0 Hz, 1H, Ar); 7.58 (d, *J* = 8.2 Hz, 2H, Ar); 7.80 (d, *J* = 8.6 Hz, 1H, Ar); 8.78 (s, 1H, Ar); 9.14 (t, *J* = 6.3 Hz, 1H, NH); 11.08 (s, 1H, OH). IR 3433, 1769, 1737 cm^−1^. Elemental analysis: calculated for C_17_H_11_Cl_2_NO_4_ (364.18)%C 56.07, %H 3.04, %N 3.85, found %C 56.07 %H 3.02, %N 3.85. *m*/*z* 365.

##### *N-(3,4-Dichlorobenzyl)-7-Hydroxy-2-Oxo-2H-Chromene-3-Carboxamide* **(23)**

Following the general procedure, the title compound was prepared starting from 3,4-dichlorobenzylamine. Yield 62% M.p. 174–175 °C. ^1^H NMR (DMSO-*d*_6_) δ 4.56 (d, *J* = 6.0 Hz, 2H, CH_2_); 6.78 (d, *J* = 2.1 Hz, 1H, Ar); 6.86 (dd, *J* = 8.7 2.1 Hz, 1H, Ar); 7.40 (m, 2H, Ar); 7.62 (m, 1H, Ar); 7.79 (d, *J* = 8.8 Hz, 1H, Ar); 8.77 (s, 1H, Ar); 9.14 (t, *J* = 5.6 Hz, 1H, NH); 10.76 (s, 1H, OH). IR 3425, 1762, 1735 cm^−1^. Elemental analysis: calculated for C_17_H_11_Cl_2_NO_4_ (364.18)%C 56.07, %H 3.04, %N 3.85, found %C 56.07 %H 3.03, %N 3.84. *m*/*z* 365.

##### *N-(2,6-Dichlorobenzyl)-7-Hydroxy-2-Oxo-2H-Chromene-3-Carboxamide* **(24)**

Following the general procedure, the title compound was prepared starting from 2,6-dichlorobenzylamine. Yield 81% M.p. 175–176 °C. ^1^H NMR (DMSO-*d*_6_) δ 4.81 (d, *J* = 5.5 Hz, 2H, CH_2_); 6.79 (d, *J* = 2.2 Hz, 1H, Ar); 6.88 (dd, *J* = 8.7 2.2 Hz, 1H, Ar); 7.39 (t, *J* = 7.8 Hz, 1H, Ar); 7.52 (d, *J* = 8.1 Hz, 2H, Ar); 7.81 (d, *J* = 8.7 Hz, 1H, Ar); 8.81 (s, 1H, Ar); 8.96 (t, *J* = 5.6 Hz, 1H, NH); 10.90 (s, 1H, OH). IR 3428, 1766, 1733 cm^−1^. Elemental analysis: calculated for C_17_H_11_Cl_2_NO_4_ (364.18)%C 56.07, %H 3.04, %N 3.85, found %C 56.08 %H 3.05, %N 3.86. *m*/*z* 365.

##### *7-Hydroxy-N-(Naphthalen-2-ylmethyl)-2-Oxo-2H-Chromene-3-Carboxamide* **(25)**

Following the general procedure, the title compound was prepared starting from 2-naphthalenemethanamine. Yield 48% M.p. 190–191 °C. ^1^H NMR (DMSO-d_6_) δ 5.01 (d, *J* = 5.68 Hz, 2H, CH_2_); 6.71 (m, 1H, Ar); 6.82 (m, 1H, Ar); 7.48 (7, *J* = 8.0 Hz, 1H, Ar); 7.55 (m, 3H, Ar); 7.77 (m, 1H, Ar); 7.87 (d, *J* = 8.2 Hz, 1H, Ar); 7.96 (d, *J* = 8.4 Hz, 1H, Ar); 8.16 (d, *J* = 8.0 Hz, 1H, Ar); 8.80 (s, 1H, Ar); 9.08 (t, *J* = 5.7 Hz, 1H, NH); 11.13 (s, 1H, OH). IR 3388, 1770, 1725 cm^−1^. Elemental analysis: calculated for C_21_H_15_NO_4_ (345.35)%C 73.03, %H 4.38, %N 4.06, found %C 73.04 %H 4.36, %N 4.06. *m*/*z* 346.

##### *7-Hydroxy-N-(4-Methoxyphenethyl)-2-Oxo-2H-Chromene-3-Carboxamide* **(26)**

Following the general procedure, the title compound was prepared starting from 4-methoxyphenethylamine. Yield 84% M.p. 202–203 °C. ^1^H NMR (DMSO-*d*_6_) δ 2.76 (t, *J* = 7.4 Hz, 2H, CH_2_); 3.51 (q, *J* = 6.9 Hz, 2H, CH_2_); 3.72 (s, 3H, OCH_3_); 6.78 (m, 1H, Ar); 6.86 (m, 3H, Ar); 7.16 (d, *J* = 7.7 Hz, 2H, Ar); 7.80 (d, *J* = 8.4 Hz, 1H, Ar); 8.67 (t, *J* = 5.0 Hz, 1H, NH); 8.77 (s, 1H, Ar); 11.04 (s, 1H, OH). IR 3405, 1778, 1722 cm^−1^. Elemental analysis: calculated for C_19_H_17_NO_5_ (339.34)%C 67.25, %H 5.05, %N 4.13, found %C 67.24 %H 5.03, %N 4.14. *m*/*z* 340.

##### *N-(3,4-Dimethoxyphenethyl)-7-Hydroxy-2-Oxo-2H-Chromene-3-Carboxamide* **(27)**

Following the general procedure, the title compound was prepared starting from 3,4-dimethoxyphenethylamine. Yield 79% M.p. 202–203 °C. ^1^H NMR (DMSO-*d*_6_) δ 2.76 (t, *J* = 7.1 Hz, 2H, CH_2_); 3.53 (q, *J* = 6.2 Hz, 2H, CH_2_); 3.71 (s, 3H, OCH_3_); 3.74 (s, 3H, OCH_3_); 6.77 (m, 2H, Ar); 6.86 (m, 3H, Ar); 7.80 (d, *J* = 8.3 Hz, 1H, Ar); 8.67 (t, *J* = 5.7 Hz, 1H, NH); 8.77 (s, 1H, Ar); 11.11 (s, 1H, OH). IR 3402, 1771, 1725 cm^−1^. Elemental analysis: calculated for C_20_H_19_NO_6_ (369.37)%C 65.03, %H 5.18, %N 3.79, found %C 65.01 %H 5.19, %N 3.78. *m*/*z* 370.

##### *N-(4-Fluorophenethyl)-7-Hydroxy-2-Oxo-2H-Chromene-3-Carboxamide* **(28)**

Following the general procedure, the title compound was prepared starting from 4-fluorophenethylamine. Yield 88% M.p. 205–206 °C. ^1^H NMR (DMSO-*d*_6_) δ 2.83 (t, *J* = 7.5 Hz, 2H, CH_2_); 3.54 (q, *J* = 7.0 Hz, 2H, CH_2_); 6.79 (d, *J* = 1.9 Hz, 1H, Ar); 6.88 (dd, *J =* 8.5 2.1 Hz 1H, Ar); 7.11 (t, *J* = 8.8 Hz, 2H, Ar); 7.28 (m, 2H, Ar); 7.81 (d, *J* = 8.4 Hz, 1H, Ar); 8.68 (t, *J* = 5.3 Hz, 1H, NH); 8.77 (s, 1H, Ar); 11.04 (s, 1H, OH). IR 3415, 1771, 1730 cm^−1^. Elemental analysis: calculated for C_18_H_14_FNO_4_ (327.31)%C 66.05, %H 4.31, %N 4.28, found %C 66.06 %H 4.30, %N 4.28. *m*/*z* 340.

##### *N-(2-Chlorophenethyl)-7-Hydroxy-2-Oxo-2H-Chromene-3-Carboxamide* **(29)**

Following the general procedure, the title compound was prepared starting from 2-chlorophenethylamine. Yield 78% M.p. 200–201 °C. ^1^H NMR (DMSO-*d*_6_) δ 2.97 (t, *J* = 7.4 Hz, 2H, CH_2_); 3.58 (q, *J* = 6.6 Hz, 2H, CH_2_); 6.79 (d, *J* = 1.6 Hz, 1H, Ar); 6.88 (dd, *J =* 8.7 1.8 Hz 1H, Ar); 7.27 (m, 2H, Ar); 7.37 (d, *J* = 7.6 Hz, 1H, Ar); 7.42 (d, *J* = 7.9 Hz, 1H, Ar); 7.80 (d, *J* = 8.5 Hz, 1H, Ar); 8.71 (t, *J* = 5.7 Hz, 1H, NH); 8.77 (s, 1H, Ar); 11.10 (s, 1H, OH). IR 3393, 1775, 1728 cm^−1^. Elemental analysis: calculated for C_18_H_14_ClNO_4_ (343.76)%C 62.89, %H 4.10, %N 4.07, found %C 66.89 %H 4.11, %N 4.08. *m*/*z* 345.

##### *N-(3-Chlorophenethyl)-7-Hydroxy-2-Oxo-2H-Chromene-3-Carboxamide* **(30)**

Following the general procedure, the title compound was prepared starting from 3-chlorophenethylamine. Yield 90% M.p. 205–206 °C. ^1^H NMR (DMSO-*d*_6_) δ 2.85 (t, *J* = 7.4 Hz, 2H, CH_2_); 3.56 (q, *J* = 6.8 Hz, 2H, CH_2_); 6.79 (d, *J* = 2.0 Hz, 1H, Ar); 6.87 (dd, *J =* 8.6 2.1 Hz 1H, Ar); 7.22 (d, *J* = 7.5 Hz, 1H, Ar); 7.27 (d, *J* = 7.5 Hz, 1H, Ar); 7.32 (m, 2H, Ar); 7.81 (d, *J* = 8.6 Hz, 1H, Ar); 8.69 (t, *J* = 5.8 Hz, 1H, NH); 8.78 (s, 1H, Ar); 11.12 (s, 1H, OH). IR 3395, 1778, 1731 cm^−1^. Elemental analysis: calculated for C_18_H_14_ClNO_4_ (343.76)%C 62.89, %H 4.10, %N 4.07, found %C 62.87 %H 4.09, %N 4.07. *m*/*z* 345.

##### *3-(4-Benzylpiperazine-1-Carbonyl)-7-Hydroxy-2H-Chromen-2-One* **(31)**

Following the general procedure, the title compound was prepared starting from benzylpiperazine. Yield 82% M.p. 208–209 °C. ^1^H NMR (DMSO-*d*_6_) δ 3.11 (m, 2H, -CH_2_); 3.19 (m, 2H, CH_2_); 3.52 (m, 2H, CH_2_); 3.78 (m, 2H, CH_2_); 4.37 (s, 2H, CH_2_); 6.74 (m, 2H, Ar); 6.89 (d, *J* = 8.2 Hz, 1H, Ar); 7.04 (m, 3H, Ar); 7.29 (m, 2H, Ar); 7.62 (d, *J* = 8.3 Hz, 1H, Ar); 8.12 (s, 1H, Ar); 10.89 (s, 1H, OH). IR 3411, 1775, 1734 cm^−1^. Elemental analysis: calculated for C_21_H_20_N_2_O_4_ (364.39)%C 69.22, %H 5.53, %N 7.69, found %C 69.22 %H 5.51, %N 7.70. *m*/*z* 365.

##### *7-Hydroxy-3-(4-Phenylpiperazine-1-Carbonyl)-2H-Chromen-2-One* **(32)**

Following the general procedure, the title compound was prepared starting from phenylpiperazine. Yield 64% M.p. 197–198 °C. ^1^H NMR (DMSO-*d*_6_) δ 3.10 (m, 2H, -CH_2_); 3.17 (m, 2H, CH_2_); 3.47 (m, 2H, CH_2_); 3.72 (m, 2H, CH_2_); 6.78 (m, 3H, Ar); 6.84 (d, *J* = 7.8 Hz, 1H, Ar); 6.86 (m, 2H, Ar); 7.21 (m, 2H, Ar); 7.57 (d, *J* = 8.1 Hz, 1H, Ar); 8.10 (s, 1H, Ar); 10.86 (s, 1H, OH). IR 3382, 1770, 1724 cm^−1^. Elemental analysis: calculated for C_20_H_18_N_2_O_4_ (350.37)%C 68.56, %H 5.18, %N 8.00, found %C 68.58 %H 5.17, %N 8.01. *m*/*z* 351.

##### *7-Hydroxy-3-(4-(m-tolyl)piperazine-1-Carbonyl)-2H-Chromen-2-One* **(33)**

Following the general procedure, the title compound was prepared starting from 4-methylphenylpiperazine. Yield 79% M.p. 202–203 °C. ^1^H NMR (DMSO-*d*_6_) δ 2.32 (s, 3H, CH_3_); 3.08 (m, 2H, CH_2_); 3.16 (m, 2H, CH_2_); 3.47 (m, 2H, CH_2_); 3.71 (m, 2H, CH_2_); 6.61 (m, 1H, Ar); 6.74 (m, 3H, Ar); 6.84 (d, *J* = 8.5 Hz, 1H, Ar); 7.09 (t, *J* = 8.4 Hz, 2H, Ar); 7.58 (d, *J* = 8.7 Hz, 1H, Ar); 8.09 (s, 1H, Ar); 10.82 (s, 1H, OH). IR 3412, 1770, 1735 cm^−1^. Elemental analysis: calculated for C_21_H_20_N_2_O_4_ (364.39)%C 69.22, %H 5.53, %N 7.69, found %C 69.21 %H 5.53, %N 7.68. *m*/*z* 365.

##### *7-Hydroxy-3-(4-(p-tolyl)piperazine-1-Carbonyl)-2H-Chromen-2-One* **(34)**

Following the general procedure, the title compound was prepared starting from 4-methylphenylpiperazine. Yield 72% M.p. 199–200 °C. ^1^H NMR (DMSO-*d*_6_) δ 2.25 (s, 3H, CH_3_); 2.79 (m, 2H, CH_2_); 2.85 (m, 2H, CH_2_); 3.48 (m, 2H, CH_2_); 3.73 (m, 2H, CH_2_); 6.75 (m, 1H, Ar); 6.82 (d, *J* = 8.1 Hz, 1H, Ar); 6.94 (d, *J* = 8.6 Hz, 2H, Ar); 7.12 (d, *J* = 8.6 Hz, 2H, Ar); 7.58 (d, *J* = 8.2 Hz, 1H, Ar); 8.11 (s, 1H, Ar); 10.74 (s, 1H, OH). IR 3402, 1778, 1732 cm^−1^. Elemental analysis: calculated for C_21_H_20_N_2_O_4_ (364.39)%C 69.22, %H 5.53, %N 7.69, found %C 69.23 %H 5.54, %N 7.69. *m*/*z* 365.

##### *3-(4-(2,3-Dimethylphenyl)piperazine-1-Carbonyl)-7-Hydroxy-2H-Chromen-2-One* **(35)**

Following the general procedure, the title compound was prepared starting from 2,3-dimethylphenylpiperazine. Yield 66% M.p. 207–208 °C. ^1^H NMR (DMSO-*d*_6_) δ 2.17 (s, 3H, CH_3_), 2.20 (s, 3H, CH_3_); 2.75 (m, 2H, CH_2_); 2.81 (m, 2H, CH_2_); 3.49 (m, 2H, CH_2_); 3.74 (m, 2H, CH_2_); 6.75 (m, 1H, Ar); 6.86 (m, 3H, Ar); 7.02 (d, *J* = 7.8 Hz, 1H, Ar); 7.59 (d, *J* = 8.2 Hz, 1H, Ar); 8.10 (s, 1H, Ar); 10.76 (s, 1H, OH). IR 3387, 1772, 1730 cm^−1^. Elemental analysis: calculated for C_22_H_22_N_2_O_4_ (378.42)%C 69.83, %H 5.86, %N 7.40, found %C 69.84 %H 5.86, %N 7.38. *m*/*z* 379.

##### *3-(4-(2,4-Dimethylphenyl)piperazine-1-Carbonyl)-7-Hydroxy-2H-Chromen-2-One* **(36)**

Following the general procedure, the title compound was prepared starting from 2,4-dimethylphenylpiperazine. Yield 62% M.p. 213–214 °C. ^1^H NMR (DMSO-*d*_6_) δ 2.20 (s, 3H, CH_3_); 2.23 (s, 3H, CH_3_); 2.76 (t, *J* = 4.2 Hz, 2H, CH_2_); 2.82 (t, *J* = 4.1 Hz, 2H, CH_2_); 3.48 (t, *J* = 4.3 Hz, 2H, CH_2_); 3.73 (t, *J* = 4.4 Hz, 2H, CH_2_); 6.76 (d, *J* = 2.2 Hz, 1H, Ar); 6.84 (dd, *J* = 8.5 2.1 Hz, 1H, Ar); 6.90 (d, *J* = 8.0 Hz, 1H, Ar); 6.94 (d, *J* = 8.2 Hz, 1H, Ar); 6.98 (m, 1H, Ar); 7.60 (d, *J* = 8.5 Hz, 1H, Ar); 8.12 (s, 1H, Ar); 10.75 (s, 1H, OH). IR 3381, 1774, 1731 cm^−1^. Elemental analysis: calculated for C_22_H_22_N_2_O_4_ (378.42)%C 69.83, %H 5.86, %N 7.40, found %C 69.83 %H 5.84, %N 7.39. *m*/*z* 379.

##### *3-(4-(2,5-Dimethylphenyl)piperazine-1-Carbonyl)-7-Hydroxy-2H-Chromen-2-One* **(37)**

Following the general procedure, the title compound was prepared starting from 2,5-dimethylphenylpiperazine. Yield 70% M.p. 224–225 °C. ^1^H NMR (DMSO-*d*_6_) δ 2.21 (s, 3H, CH_3_); 2.24 (s, 3H, CH_3_); 2.79 (t, *J* = 4.3 Hz, 2H, CH_2_); 2.85 (t, *J* = 4.3 Hz, 2H, CH_2_); 3.48 (t, *J* = 4.4 Hz, 2H, CH_2_); 3.73 (t, *J* = 4.4 Hz, 2H, CH_2_); 6.76 (d, *J* = 2.0 Hz, 1H, Ar); 6.78 (d, *J* = 7.5 Hz, 1H, Ar); 6.83 (m, 2H, Ar); 7.04 (d, *J* = 7.5 Hz, 1H, Ar); 7.60 (d, *J* = 8.5 Hz, 1H, Ar); 8.12 (s, 1H, Ar); 10.91 (s, 1H, OH). IR 3382, 1775, 1727 cm^−1^. Elemental analysis: calculated for C_22_H_22_N_2_O_4_ (378.42)%C 69.83, %H 5.86, %N 7.40, found %C 69.84 %H 5.88, %N 7.41. *m*/*z* 379.

##### *3-(4-(3,4-Dimethylphenyl)piperazine-1-Carbonyl)-7-Hydroxy-2H-Chromen-2-One* **(38)**

Following the general procedure, the title compound was prepared starting from 3,4-dimethylphenylpiperazine. Yield 52% M.p. 222–223 °C. ^1^H NMR (DMSO-*d*_6_) δ 2.11 (s, 3H, CH_3_); 2.17 (s, 3H, CH_3_); 3.03 (t, *J* = 4.4 Hz, 2H, CH_2_); 3.11 (t, *J* = 4.5 Hz, 2H, CH_2_); 3.47 (t, *J* = 4.3 Hz, 2H, CH_2_); 3.72 (t, *J* = 4.2 Hz, 2H, CH_2_); 6.67 (dd, *J* = 8.3, 2.4 Hz, 1H, Ar); 6.77 (m, 2H, Ar); 6.84 (dd, *J* = 8.6, 2.2 Hz, 1H, Ar); 6.97 (d, *J* = 8.3 Hz, 1H, Ar); 7.60 (d, *J* = 8.5 Hz, 1H, Ar); 8.11 (s, 1H, Ar); 10.89 (s, 1H, OH). IR 3384, 1779, 1732 cm^−1^. Elemental analysis: calculated for C_22_H_22_N_2_O_4_ (378.42)%C 69.83, %H 5.86, %N 7.40, found %C 69.82 %H 5.86, %N 7.38. *m*/*z* 379.

##### *3-(4-(3,5-Dimethylphenyl)piperazine-1-Carbonyl)-7-Hydroxy-2H-Chromen-2-One* **(39)**

Following the general procedure, the title compound was prepared starting from 3,5-dimethylphenylpiperazine. Yield 62% M.p. 224–225 °C. ^1^H NMR (DMSO-*d*_6_) δ 2.20 (s, 6H, CH_3_); 3.08 (t, *J* = 4.5 Hz, 2H, CH_2_); 3.15 (t, *J* = 4.2 Hz, 2H, CH_2_); 3.47 (t, *J* = 4.1 Hz, 2H, CH_2_); 3.71 (t, *J* = 4.3 Hz, 2H, CH_2_); 6.47 (m, 1H, Ar); 6.57 (m, 2H, Ar); 6.76 (d, *J* = 1.9 Hz, 1H, Ar); 6.84 (dd, *J* = 8.7, 2.1 Hz, 1H, Ar); 7.60 (d, *J* = 8.7 Hz, 1H, Ar); 8.11 (s, 1H, Ar); 10.79 (s, 1H, OH). IR 3372, 1776, 1730 cm^−1^. Elemental analysis: calculated for C_22_H_22_N_2_O_4_ (378.42)%C 69.83, %H 5.86, %N 7.40, found %C 69.85 %H 5.87, %N 7.39. *m*/*z* 379.

##### *7-Hydroxy-3-(4-(2-Nitrophenyl)piperazine-1-Carbonyl)-2H-Chromen-2-One* **(40)**

Following the general procedure, the title compound was prepared starting from 2-nitrophenylpiperazine. Yield 65% M.p. 212–213 °C. ^1^H NMR (DMSO-*d*_6_) δ 2.99 (t, *J* = 4.1 Hz, 2H, CH_2_); 3.07 (t, *J* = 4.3 Hz, 2H, CH_2_); 3.48 (t, *J* = 4.0 Hz, 2H, CH_2_); 3.71 (t, *J* = 4.1 Hz, 2H, CH_2_); 6.76 (m, 1H, Ar); 6.84 (d, *J* = 7.8 Hz, 1H, Ar); 7.18 (t, *J* = 7.2 Hz, 1H, Ar); 7.36 (d, *J* = 7.8 Hz, 1H, Ar); 7.60 (m, 2H, Ar); 7.83 (d, *J* = 8.4 Hz, 1H, Ar); 8.12 (s, 1H, Ar); 10.85 (s, 1H, OH). IR 3400, 1769, 1733 cm^−1^. Elemental analysis: calculated for C_20_H_17_N_3_O_6_ (395.37)%C 60.76, %H 4.33, %N 10.63, found %C 60.78 %H 4.31, %N 10.63. *m*/*z* 396.

##### *7-Hydroxy-3-(4-(3-Nitrophenyl)piperazine-1-Carbonyl)-2H-Chromen-2-One* **(41)**

Following the general procedure, the title compound was prepared starting from 3-nitrophenylpiperazine. Yield 47% M.p. 215–216 °C. ^1^H NMR (DMSO-*d*_6_) δ 3.28 (t, *J* = 4.2 Hz, 2H, CH_2_); 3.37 (t, *J* = 4.1 Hz, 2H, CH_2_); 3.53 (t, *J* = 4.2 Hz, 2H, CH_2_); 3.76 (t, *J* = 4.2 Hz, 2H, CH_2_); 6.77 (m, 1H, Ar); 6.84 (d, *J* = 7.7 Hz, 1H, Ar); 7.41 (d, *J* = 8.1 Hz, 1H, Ar); 7.49 (t, *J* = 7.7 Hz, 1H, Ar); 7.60 (m, 2H, Ar); 7.67 (m, 1H, Ar); 8.13 (s, 1H, Ar); 10.89 (s, 1H, OH). IR 3405, 1772, 1730 cm^−1^. Elemental analysis: calculated for C_20_H_17_N_3_O_6_ (395.37)%C 60.76, %H 4.33, %N 10.63, found %C 60.76 %H 4.32, %N 10.62. *m*/*z* 396.

##### *7-Hydroxy-3-(4-(4-Nitrophenyl)piperazine-1-Carbonyl)-2H-Chromen-2-One* **(42)**

Following the general procedure, the title compound was prepared starting from 4-nitrophenylpiperazine. Yield 52% M.p. 217–218 °C. ^1^H NMR (DMSO-*d*_6_) δ 3.49 (m, 2H, CH_2_); 3.54 (m, 2H, CH_2_); 3.58 (m, 2H, CH_2_); 3.73 (m, 2H, CH_2_); 6.77 (m, 1H, Ar); 6.84 (d, *J* = 7.2 Hz, 1H, Ar); 7.03 (d, *J* = 7.5 Hz, 2H, Ar); 7.60 (d, *J* = 8.2 Hz, 1H, Ar); 8.08 (d, *J* = 6.7 Hz, 1H, Ar); 8.14 (s, 1H, Ar); 11.01 (s, 1H, OH). IR 3402, 1774, 1731 cm^−1^. Elemental analysis: calculated for C_20_H_17_N_3_O_6_ (395.37)%C 60.76, %H 4.33, %N 10.63, found %C 60.77 %H 4.32, %N 10.64. *m*/*z* 396.

##### *7-Hydroxy-3-(4-(3-Methoxyphenyl)piperazine-1-Carbonyl)-2H-Chromen-2-One* **(43)**

Following the general procedure, the title compound was prepared starting from 3-methoxyphenylpiperazine. Yield 58% M.p. 204–206 °C. ^1^H NMR (DMSO-*d*_6_) δ 3.10 (m, 2H, CH_2_); 3.18 (m, 2H, CH_2_); 3.47 (m, 2H, CH_2_); 3.70 (s, 5H, CH_2_ and OCH_3_); 6.38 (m, 1H, Ar); 6.41 (m, 1H, Ar); 6.52 (d, *J* = 7.9 Hz, 1H, Ar); 6.76 (s, 1H, Ar); 6.83 (d, *J* = 7.8 Hz, 1H, Ar); 6.86 (t, *J* = 8.1 Hz, 1H, Ar); 7.11 (d, *J* = 8.2 Hz, 1H, Ar); 7.58 (d, *J* = 8.0 Hz, 1H, Ar); 8.10 (s, 1H, Ar); 10.89 (s, 1H, OH). IR 3398, 1775, 1729 cm^−1^. Elemental analysis: calculated for C_21_H_20_N_2_O_5_ (380.39)%C 66.31, %H 5.30, %N 7.36, found %C 66.30 %H 5.29, %N 7.35. *m*/*z* 381.

##### *7-Hydroxy-3-(4-(4-Methoxyphenyl)piperazine-1-Carbonyl)-2H-Chromen-2-One* **(44)**

Following the general procedure, the title compound was prepared starting from 4-methoxyphenylpiperazine. Yield 64% M.p. 200–201 °C. ^1^H NMR (DMSO-*d*_6_) δ 2.96 (m, 2H, CH_2_); 3.03 (m, 2H, CH_2_); 3.46 (m, 2H, CH_2_); 3.67 (s, 3H, OCH_3_); 3.72 (m, 2H, CH_2_); 6.76 (m, 1H, Ar); 6.81 (d, *J* = 7.9 Hz, 1H, Ar); 6.82 (m, 3H, Ar); 6.89 (d, *J* = 8.3 Hz, 2H, Ar); 7.57 (d, *J* = 8.4 Hz, 1H, Ar); 8.09 (s, 1H, Ar); 10.74 (s, 1H, OH). IR 3392, 1776, 1725 cm^−1^. Elemental analysis: calculated for C_21_H_20_N_2_O_5_ (380.39)%C 66.31, %H 5.30, %N 7.36, found %C 66.33 %H 5.30, %N 7.37. *m*/*z* 381.

##### *3-(4-(4-Trifluoromethylphenyl)piperazine-1-Carbonyl)-7-Hydroxy-2H-Chromen-2-One* **(45)**

Following the general procedure, the title compound was prepared starting from 4-trifluoromethylphenylpiperazine. Yield 74% M.p. 200–201 °C. ^1^H NMR (DMSO-*d*_6_) δ 3.42 (m, 2H, CH_2_); 3.48 (m, 2H, CH_2_); 3.56 (m, 2H, CH_2_); 3.64 (m, 2H, CH_2_); 6.77 (m, 1H, Ar); 6.84 (d, *J* = 7.6 Hz, 1H, Ar); 7.56 (d, *J* = 8.6 Hz, 2H, Ar); 7.67 (m, 3H, Ar); 8.10 (s, 1H, Ar); 10.81 (s, 1H, OH). IR 3402, 1770, 1713 cm^−1^. Elemental analysis: calculated for C_21_H_17_F_3_N_2_O_4_ (418.37)%C 60.29, %H 4.10, %N 6.70, found %C 60.30 %H 4.10, %N 6.69. *m*/*z* 419.

### 3.2. Docking Methods

Docking simulations were performed by means of GOLD (2024.1 CSD Release) [28] using the crystal structures of *h*-CA IX (PDB ID 3IAI) [18] and *h*-CA XII (PDB ID 1JD0) [29] in complex with AAZ. Protein and ligands structures were prepared as detailed in our previous paper [23]. The binding site was defined based on the crystallographic pose of the *trans*-2-hydroxy-cinnamic acid into hCA II (PDB ID 5BNL) active site [30], including all residues within 6 Å from the native ligand. Ten poses were computed for each ligand and ranked according to ChemPLP scoring function. The top ranked docking pose was selected for analysis and representation.

### 3.3. Cell Cultures

Human bronchial epithelial (BEAS-2B; CRL-3588) and human lung carcinoma (A549; CCL-185) cell lines were obtained from ATCC^®^. BEAS and A549 cells were maintained in complete RPMI 1640 (Merck, Darmstadt, Germany) at 37 °C and 5% CO_2_. The medium was supplemented with 10% heat-inactivated fetal bovine serum (FBS), 1% penicillin/streptomycin and 1% sodium pyruvate.

### 3.4. Cell Treatment

Cells were seeded at different densities according to the different experimental techniques performed (MTT assay and cell cycle analysis) and left to adhere for 24 h. Next, the medium was removed, and cells were treated with tested compounds, dissolved in dimethyl sulfoxide (DMSO) to achieve concentrations ranging from 0 µM (untreated control = CTRL) to 150 µM. The final DMSO concentration was kept below 0.3%. Cell viability was evaluated at up to 72 h post-treatment. Images were acquired 48 h after treatments by means of a light phase-contrast microscope equipped with a camera (Leica). Images were acquired and analyzed by the Leica Application Suite LAS EZ version 3.4 (Leica, Wetzlar, Germany).

### 3.5. Cell Viability (MTT Assay)

BEAS and A549 cells were seeded in 96-well culture-treated plates (Falcon^®^, Corning Incorporated, Brooklyn, NY, USA) at a density of 0.5 × 10^4^ cells/well. Untreated cells were set as the experimental control, representing 100% of cell viability. Exposure times varied from 24 up to 72 h. At the established time points (24, 48 and 72 h), the exposure medium was replaced with fresh medium containing 0.5 mg/mL 3-(4,5-dimethylthiazol-2-yl)-2,5-diphenyltetrazolium bromide (MTT) (Merck, Darmstadt, Germany) and processed as previously reported [31]. The optical density in each well was measured at a wavelength of 540 nm using a spectrophotometer (Thermo Fisher Scientific, Waltham, MA, USA). Each experiment was performed in triplicate per experimental conditions (n = 3).

### 3.6. Cytotoxicity Assay (LDH Test)

To assess cytotoxicity in A549 cells after their treatment with the tested compounds, the amount of lactate dehydrogenase released into the culture medium was quantified using the CytoTox 96 non-radioactive cytotoxicity assay (Promega, Madison, WI, USA) according to the manufacturer’s instructions [32]. Cell supernatants were collected from the same cultures processed for the MTT assay after 24 h of treatment and the optical density values obtained were normalized to those measured from the MTT assay.

### 3.7. Cell Cycle Analysis

A549 cells were seeded in 12-well plates at a density of 0.5 × 105 cells/well and treated for 6 and 24 h as previously described. At each time point, cells were detached using StemPro™ Accutase™, pelleted by centrifugation, and fixed overnight at 4 °C in 70% *v*/*v* cold ethanol. After fixation, cells were processed as described elsewhere [33]. The PI fluorescence was detected by a flow cytometer equipped with a 488 nm laser (CytoFlex flow cytometer, Beckman Coulter, CA, USA) in the FL-3 channel. A minimum of 10,000 events/sample were acquired and analyzed with the CytExpert Software, version 2.3 (Beckman Coulter, CA, USA), and the percentages of cells in the G1, S, or G2 phase of the cell cycle were calculated using the ModFit LT™ Software, version 5.0 (Verity Software House, Topsham, ME, USA).

### 3.8. Statistical Analysis

Statistics were performed using two-way analysis of variance (ANOVA) followed by Dunnet and Tukey’s multiple comparison tests by means of the Prism 8.0 software (GraphPad, San Diego, CA, USA). Results are presented as mean values ± standard deviations. Values of *p* ≤ 0.05 were considered statistically significant.

## Figures and Tables

**Table 1:** Inhibition activity of compounds **4**−**25** on *h*-CA I, *h*-CA II, *h*-CA IX, and *h*-CA XII, using AAZ as reference compound.

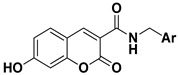					
Compound	Ar	K_i_ (µM) *			
		*h*-CA I	*h*-CA II	*h*-CA IX	*h*-CA XII
**4**	phenyl	>100	>100	42.2	7.5
**5**	2-CH_3_ phenyl	>100	>100	25.4	35.5
**6**	3-CH_3_ phenyl	>100	>100	82.5	9.3
**7**	4-CH_3_ phenyl	>100	>100	33.7	8.8
**8**	2-OCH_3_ phenyl	>100	>100	77.4	70.2
**9**	4-OCH_3_ phenyl	>100	>100	53.8	9.1
**10**	3-OCH_3_ phenyl	>100	>100	63.1	73.1
**11**	4-NO_2_ phenyl	>100	>100	75.7	49.8
**12**	3-NO_2_ phenyl	>100	>100	64.7	55.2
**13**	4-F phenyl	>100	>100	86.9	70.5
**14**	3-F phenyl	>100	>100	73.2	57.6
**15**	2-CF_3_ phenyl	>100	>100	50.0	31.5
**16**	3-CF_3_ phenyl	>100	>100	77.1	64.4
**17**	4-CF_3_ phenyl	>100	>100	83.7	54.1
**18**	4-Br phenyl	>100	>100	60.7	26.1
**19**	2-Cl phenyl	>100	>100	19.3	64.9
**20**	3-Cl phenyl	>100	>100	33.9	42.6
**21**	4-Cl phenyl	>100	>100	80.7	39.7
**22**	2,4-diCl phenyl	>100	>100	22.0	84.1
**23**	3,4-diCl phenyl	>100	>100	46.2	21.0
**24**	2,6-diCl phenyl	>100	>100	85.6	75.1
**25**	naphthyl	>100	>100	18.3	9.6
**AAZ**	-	0.25	0.012	0.025	0.006

* K_i_ values are reported as means of three independent experiments. Errors are in the range of ±5–10% of the reported values.

**Table 2:** Inhibition activity of compounds **26**−**30** on *h*-CA I, *h*-CA II, *h*-CA IX, and *h*-CA XII, using AAZ as reference compound.

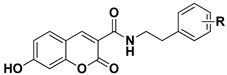					
Compound	R	K_i_ (µM) *			
		*h*-CA I	*h*-CA II	*h*-CA IX	*h*-CA XII
**26**	4-OCH_3_	>100	>100	80.8	57.7
**27**	3,4-diOCH_3_	>100	>100	84.7	50.8
**28**	4-F	>100	>100	80.4	66.9
**29**	2-Cl	>100	>100	5.5	43.1
**30**	3-Cl	>100	>100	7.5	55.8
**AAZ**		0.25	0.012	0.025	0.006

* K_i_ values are reported as means of three independent experiments. Errors are in the range of ±5–10% of the reported values.

**Table 3:** Inhibition activity of compounds **31**−**45** on *h*-CA I, *h*-CA II, *h*-CA IX, and *h*-CA XII, using AAZ as reference compound.

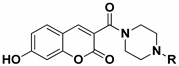					
Compound	R	K_i_ (µM) *			
		*h*-CA I	*h*-CA II	*h*-CA IX	*h*-CA XII
**31**	benzyl	>100	>100	84.1	59.5
**32**	phenyl	>100	>100	49.3	9.1
**33**	3-CH_3_ phenyl	>100	>100	89.3	40.5
**34**	4-CH_3_ phenyl	>100	>100	83.2	56.0
**35**	2,3-diCH_3_ phenyl	>100	>100	82.2	41.4
**36**	2,4-diCH_3_ phenyl	>100	>100	78.1	69.7
**37**	2,5-diCH_3_ phenyl	>100	>100	75.9	63.4
**38**	3,4-diCH_3_ phenyl	>100	>100	70.3	9.2
**39**	3,5-diCH_3_ phenyl	>100	>100	68.1	46.8
**40**	2-NO_2_ phenyl	>100	>100	85.2	63.8
**41**	3-NO_2_ phenyl	>100	>100	25.4	47.5
**42**	4-NO_2_ phenyl	>100	>100	30.1	62.8
**43**	3-OCH_3_ phenyl	>100	>100	40.4	79.9
**44**	4-OCH_3_ phenyl	>100	>100	32.8	22.5
**45**	4-CF_3_ phenyl	>100	>100	22.6	8.4
**AAZ**		0.25	0.012	0.025	0.006

* K_i_ values are reported as means of three independent experiments. Errors are in the range of ±5–10% of the reported values.

**Table 4:** Compounds, their IC_50_ values on BEAS and A549 cells, and their selectivity index (IC_50_ BEAS/IC_50_ A549 ratio). *IC_50_ values were determined using nonlinear regression analysis in GraphPad and are presented as predictive estimates.

Compound	IC_50_ BEAS (µM)	IC_50_ A549 (µM)	SI
**4**	344.90 ± 3.14	159.30 ± 1.37	2.165
**5**	181.20 ± 1.20	248.20 ± 1.57	0.730
**6**	117.90 ± 1.08	90.05 ± 1.07	1.309
**7**	157.80 ± 1.07	85.78 ± 1.13	1.840
**9**	115.90 ± 1.22	68.20 ± 1.11	1.699
**19**	40.71 ± 1.10	32.03 ± 1.22	1.271
**22**	95.82 ± 1.07	83.34 ± 1.08	1.150
**23**	73.39 ± 1.10	39.50 ± 1.06	1.858
**25**	82.35 ± 1.10	46.36 ± 1.10	1.776
**29**	77.49 ± 1.08	55.89 ± 1.12	1.386
**30**	87.42 ± 1.06	59.68 ± 1.07	1.465
**32**	399.70 ± 1.68	156.90 ± 1.12	2.547
**38**	187.40 ± 1.27	50.02 ± 1.28	3.747
**44**	203.50 ± 1.19	107.80 ± 1.28	1.888
**45**	53.85 ± 1.07	25.69 ± 1.14	2.096

## Data Availability

Data is contained within the article and Supplementary Material.

## Supplementary Materials

The following supporting information can be downloaded at https://www.mdpi.com/article/10.3390/ph18030372/s1, Figure S1: Cell cycle analysis of A549 cells exposed to increasing concentrations; Figure S2: Cell cycle analysis of A549 cells exposed to increasing concentrations of tested coumarinamides; Figure S3: ^1^HNMR spectra of representative coumarinamides **7**, **9**, **23** and **38**.

## Abbreviations

The following abbreviations are used in this manuscript:

*h*-CA	Human carbonic anhydrase
MeCN	Acetonitrile
HIF	Hypoxia-inducible factor
EDCI	1-(3-dimethylaminopropyl)-3-ethylcarbodiimide hydrochloride
HOBt	1-hydroxybenzotriazole
AAZ	Acetazolamide
A549	Bronchial adenocarcinoma cells
BEAS-2B	Normal bronchial epithelial cells
LDH	Lactate dehydrogenase
