# Supplementary material for: Investigation on Human Carbonic Anhydrase IX and XII Inhibitory Activity and A549 Antiproliferative Activity of a New Class of Coumarinamides
Source: Pharmaceuticals (Basel). 2025 Mar 5;18(3):372. doi: 10.3390/ph18030372 (PMC11944513; doi:10.3390/ph18030372)
Supplement: Supplementary file 1 [file pharmaceuticals-18-00372-s001.zip › pharmaceuticals-3492261-supplementary.pdf]

Investigation on Human Carbonic Anhydrase IX and XII inhibitory activity and A549 antiproliferative activity of new class of coumarinamides

Davide Moi, [1] Simone Carradori,[2] Marialucia Gallorini,[2], Noemi Mencarelli,[2] Alberto Deplano, [1] Andrea Angeli,[3] Serena Vittorio,[4] Claudiu T. Supuran,[3] and Valentina Onnis [1,*]

[1] Department of Life and Environmental Sciences, Unit of Pharmaceuitical, Pharmacological and Nutraceutical Sciences, University of Cagliari, Cittadella Universitaria di Monserrato, 09042 Monserrato, Cagliari, Italy; davide.moi@unica.it, vonnis@unica.it ; alberto.deplano00@gmail.com

[2] Department of Pharmacy, "G. d'Annunzio" University of Chieti-Pescara, via dei Vestini 31, 66100 Chieti, Italy; simone.carradori@unich.it; marialucia.gallorini@unich.it; noemi.mencarelli@phd.unich.it

[3] NEUROFARBA Department, Sezione di Scienze Farmaceutiche, University of Florence, Via Ugo Schiff 6, 50019, Sesto Fiorentino, Florence, Italy.; andrea.angeli@unifi.it; claudiu.supuran@unifi.it

[4] Dipartimento di Scienze Farmaceutiche, Università degli Studi di Milano, Via Mangiagalli, 25, I-20133 Milano, Italy: serena.vittorio@unimi.it

* Correspondence: vonnis@unica.it; Tel.: (optional; include country code; if there are multiple corresponding authors, add author initials)

Supporting informations content;

Figure S1: Viability of BEAS and A549 cells exposed to increasing concentrations of tested coumaninamides
Cell cycle analysis of A549 cells exposed to increasing concentrations
Figure S2: Cell cycle analysis of A549 cells exposed to increasing concentrations of tested coumarinamides
Figure S3: ^{1}HNMR spectra of representative coumarinamides **7**, **9**, **23** and **38**

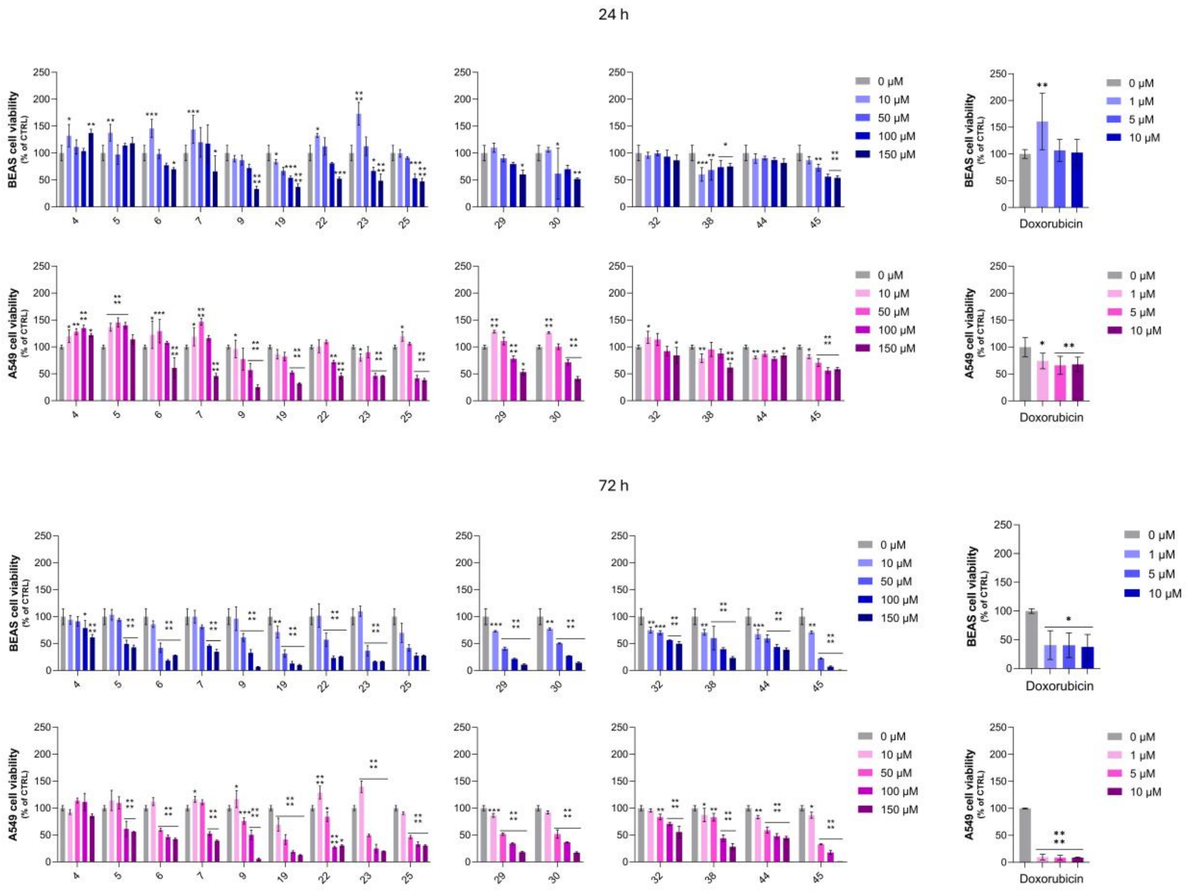

Figure S1. Cell viability of BEAS and A549 cells exposed to increasing concentrations (0-150 μM) of selected compounds (**4**, **5**, **6**, **7**, **9**, **19**, **22**, **23**, **25**, **29**, **30**, **32**, **38**, **40**, **45**) and doxorubicin for 24 and 72 hours. The bar graphs represent cell viability percentages. The untreated control (CTRL = 0 μM) is set as the 100%. Data are presented as means ± standard deviations obtained from one experiment in triplicates (n = 3). * = p < 0.01, ** = p < 0.001, *** = p < 0.0001, **** = p <0.00001 comparing treated to the untreated control.

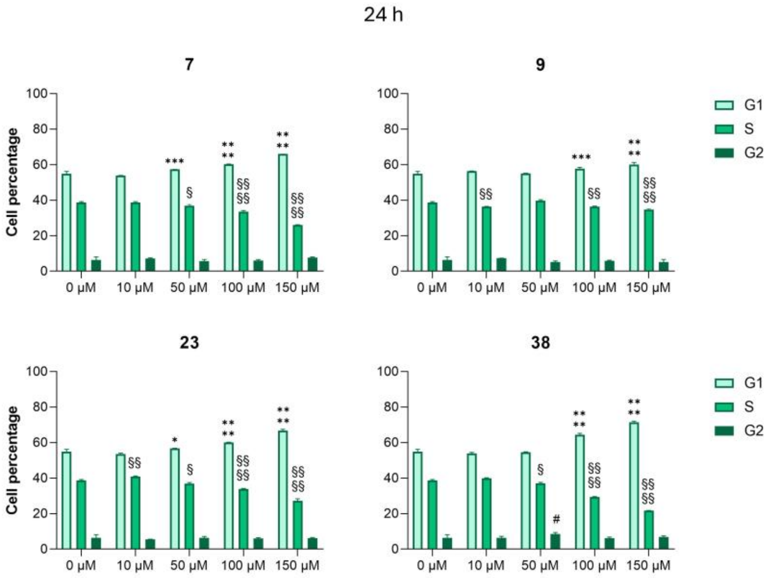

Figure S2. Cell cycle analysis in A549 cells exposed to increasing concentrations (0-150 μM) of selected compounds (**7**, **9**, **23** and **38**) after 48 hours. Data are presented as means ± standard deviations from three independent experiments (n = 3). Bars highlight cell percentages in the various phases of cell cycle (G1, S, and G2) of A549. * = $p < 0.01$, *** = $p < 0.0001$, **** = $p < 0.00001$ comparing treated to the untreated control (G1 phase). § = $p < 0.01$, §§ = $p < 0.001$, §§§§ = $p < 0.00001$ comparing treated to the untreated control (S phase). # = $p < 0.01$ comparing treated to the untreated control (G2 phase).

^{1}H NMR spectra of final compounds **7**, **9**, **23**, **38**

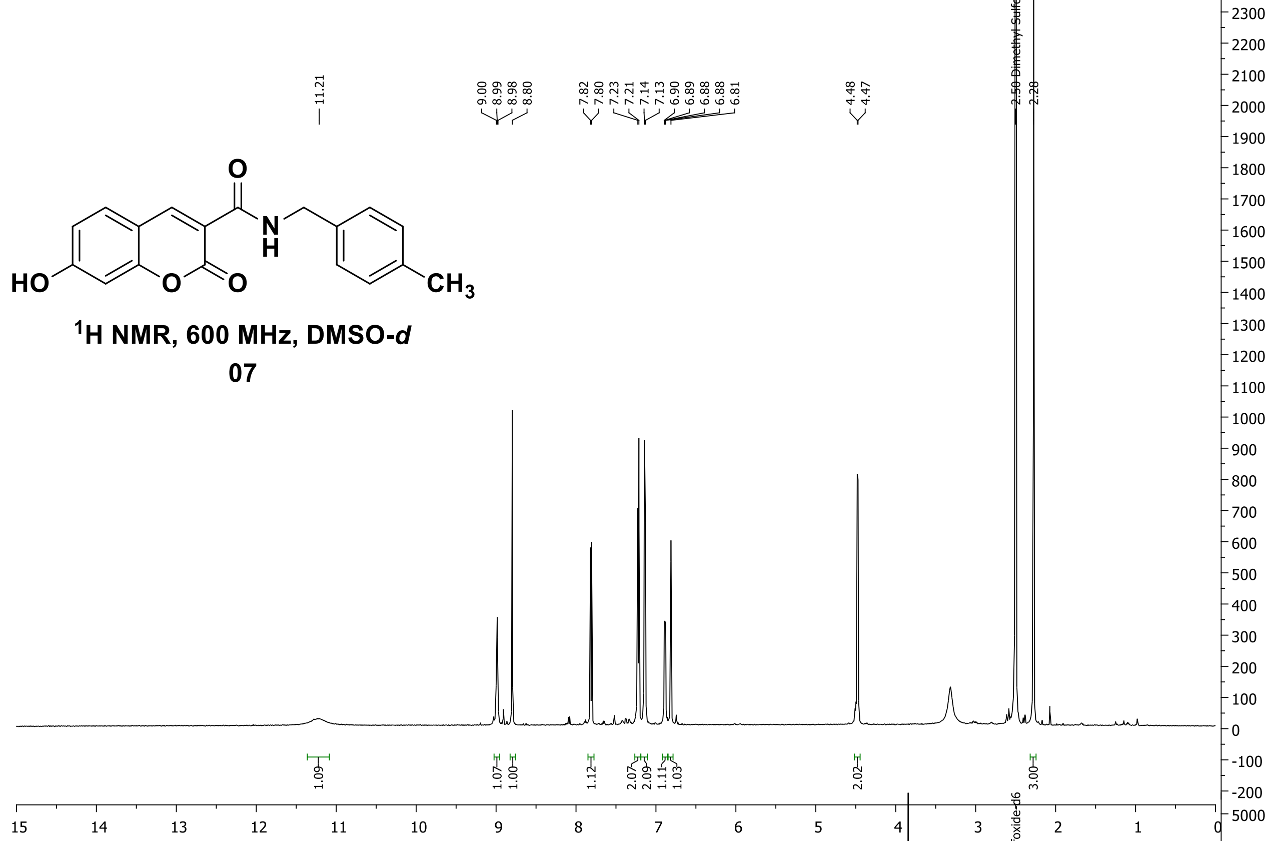

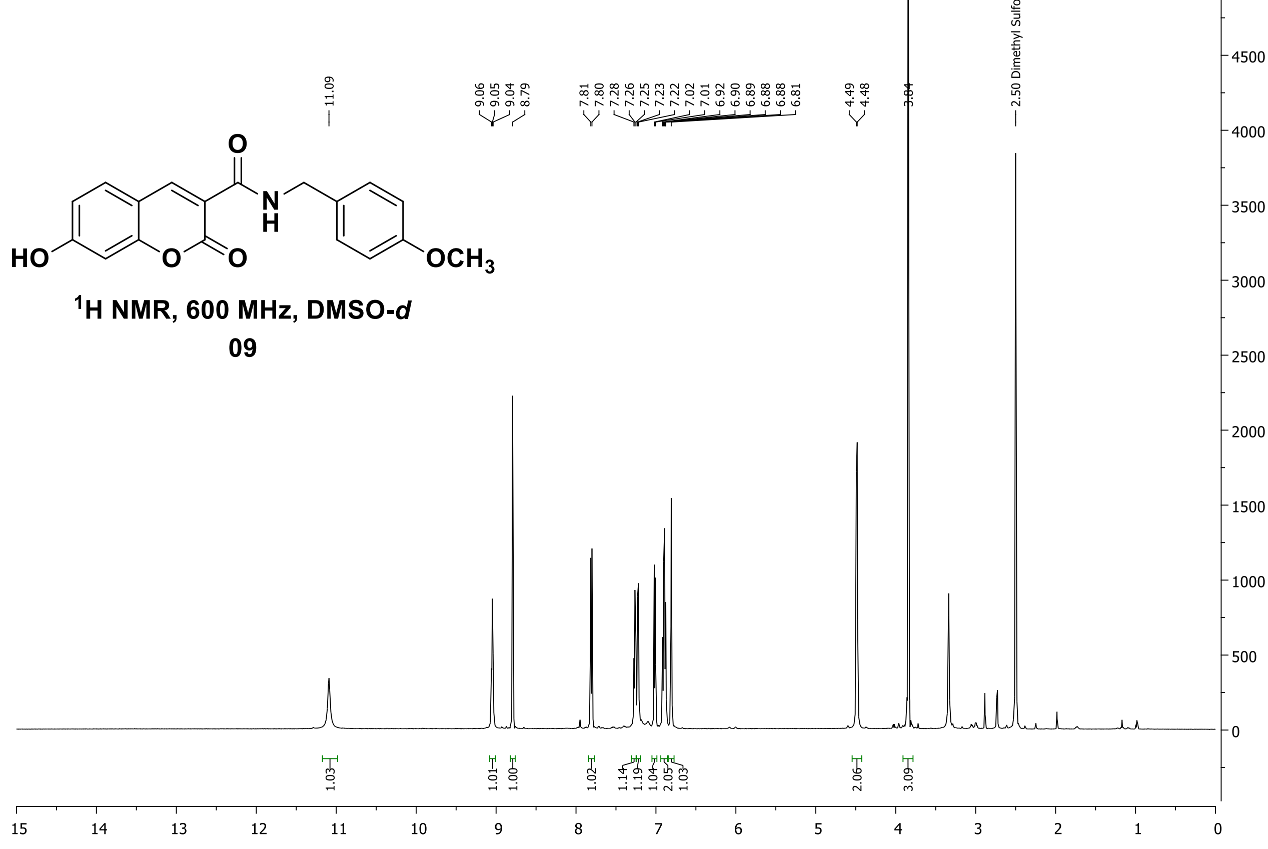

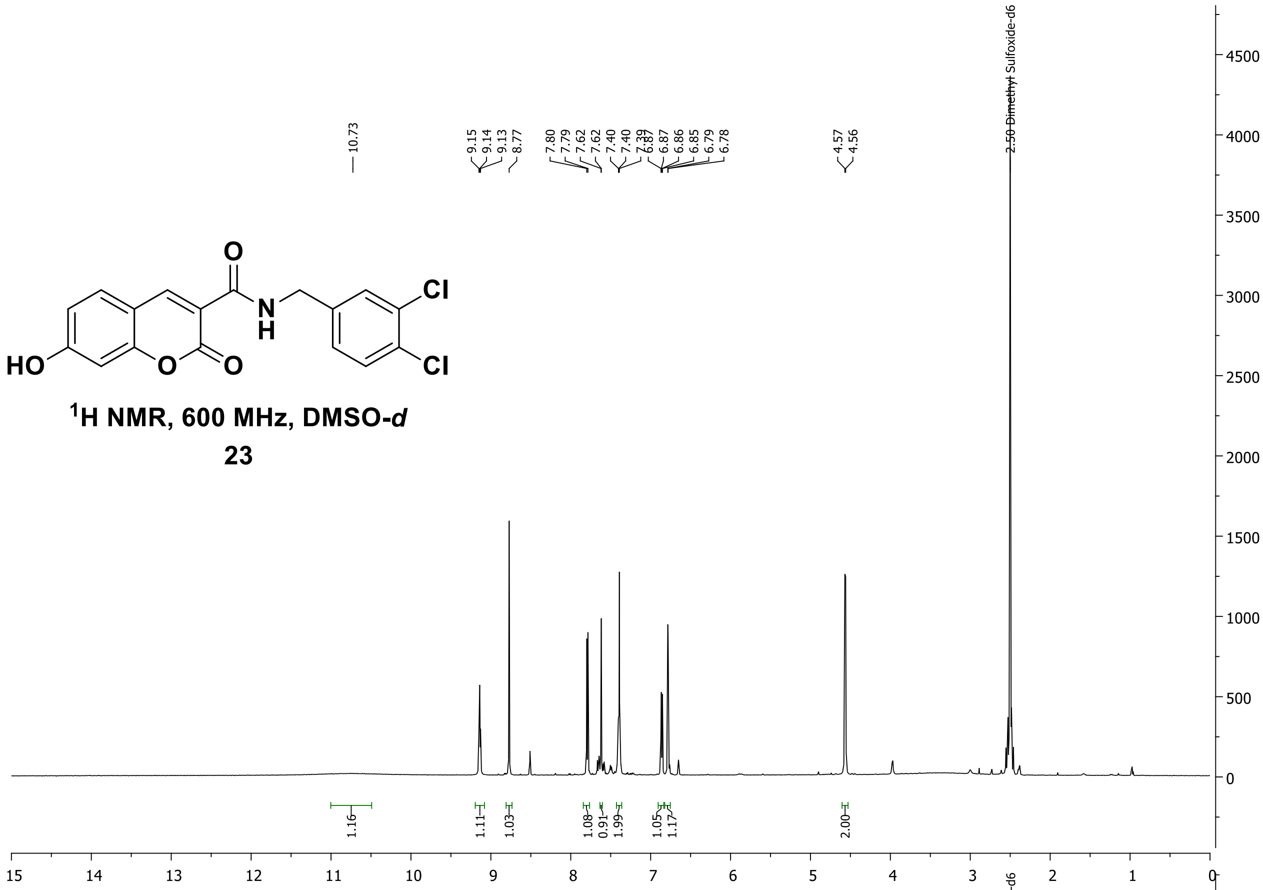

^{1}H NMR, 600 MHz, DMSO-*d*

23

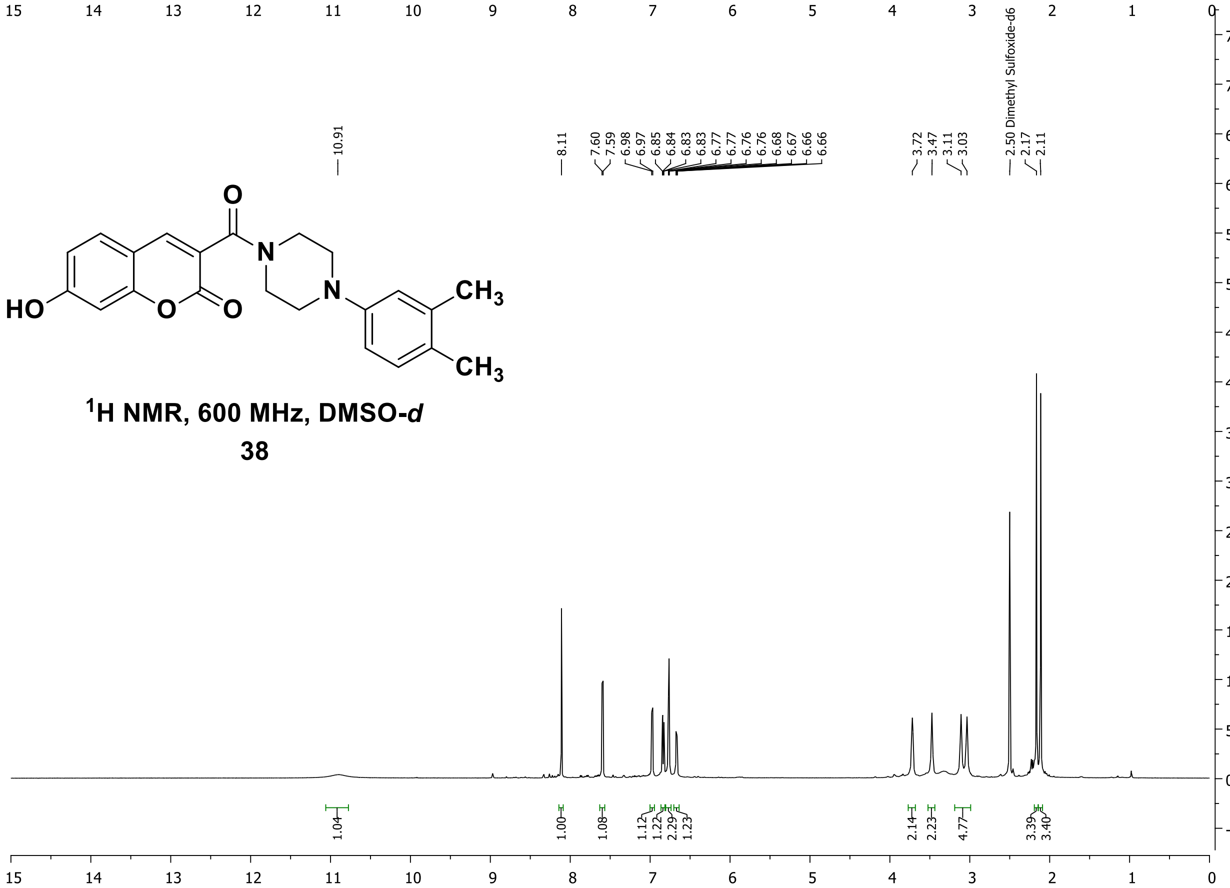

^{1}H NMR, 600 MHz, DMSO-*d*

38
